# The Gibbs and Butler Equations and the Surface Activity of Dilute Aqueous Solutions of Strong and Weak Linear Polyelectrolyte–Surfactant Mixtures: The Roles of Surface Composition and Polydispersity

**DOI:** 10.1021/acs.jpcb.4c03541

**Published:** 2024-08-14

**Authors:** Jeffrey Penfold, Robert K. Thomas

**Affiliations:** † Rutherford-Appleton Laboratory, Chilton, Didcot, Oxfordshire OX11 0RA, U.K.; ‡ Physical and Theoretical Chemistry Laboratory, South Parks Road, Oxford OX1 3QZ, U.K.

## Abstract

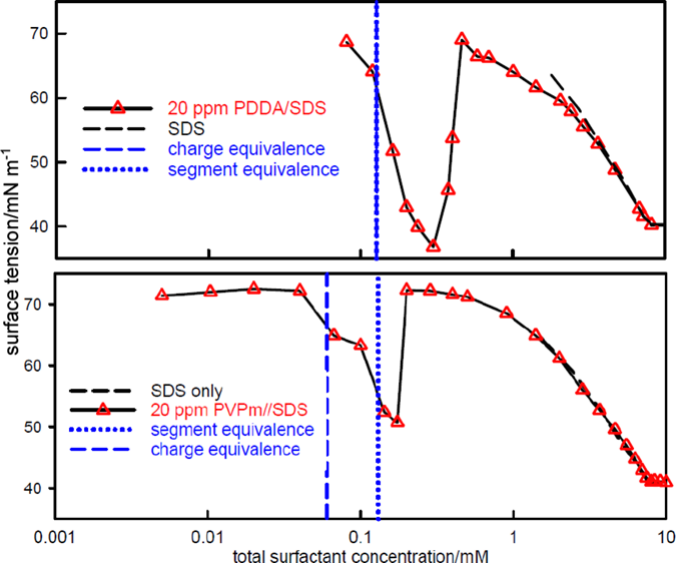

In a previous paper, we applied a combination of direct measurements of both surface tension and surface excess in conjunction with the Gibbs equation to explain features of the adsorption and surface tension of mixtures of surfactants and strong linear polyelectrolytes at the air–water interface. This paper extends that model by including (i) the restrictions of the Butler equation for the behavior of the surface tension of mixed systems and (ii) the surface behavior of surfactant and linear weak polyelectrolyte mixtures, for which the inclusion of measurements of the surface excess and composition is shown to be particularly important. In addition, a closer examination of earlier data at higher concentrations provides evidence that the surface layering that is often observed in polyelectrolyte–surfactant systems is also an average equilibrium phenomenon and is driven by particular aggregation patterns that occur in some systems and not in others. Although the successful application of the Gibbs and Butler equations indicates that strong polyelectrolyte–surfactant systems can be described in terms of an average equilibrium over wide ranges of concentration, we have identified two concentration ranges where polydispersity in either polyelectrolyte molecular weight or composition results in significant time dependence of the surface behavior.

## Introduction

The addition of ionic surfactants to dilute aqueous solutions of oppositely charged polyelectrolytes often reduces the surface tension at surfactant concentrations an order of magnitude lower than surfactants on their own.^1^ This strong interfacial activity of polyelectrolyte–surfactant (PE–S) solutions makes them valuable for many applications, from cleaning and coating surfaces^2^ to more recent biomedical applications.^3−5^ Their surface behavior is, however, complicated by (i) unusual sharp increases in the surface tension with increasing surfactant concentration,^6,7^ (ii) solute surface compositions different from those of the solutes,^6,8^ (iii) occurrence of complex surface structures at higher concentrations for some PE–S but not for others,^8,9^ and (iv) time variations of the surface tension, e.g., ref (10). These features complicate the interpretation of the surface and bulk behavior of PE–S systems as well as their applications.^11^

The surface tension and composition of an air–water (A–W) surface at equilibrium must obey the Gibbs equation and, if there is more than one solute, also the Butler equation. The test of equilibration of such solutions is whether or not these equations are satisfied. This requires independent measurements of the surface tension as a function of the bulk concentrations/activities and of the corresponding variation of the amount of each component at the A–W interface, all over a wide range of concentration. Although several direct methods of measuring surface excess at the A–W interface have been attempted, they mostly involve uncertain assumptions (for a recent review see ref (12)). The least uncertain and most versatile method has so far been neutron reflection (NR), especially when the surface contains more than one solute, as in PE–S systems.^13^ Alternative criteria for equilibration have been either that the surface tension reaches a steady state in a certain time (e.g., refs (14,15)) or that it is reproduced by different methods of measurement and/or surface preparation.

PE–S systems are slow to equilibrate, especially at the concentrations in which they are often used, and there has therefore been a strong focus on their non-equilibrium aspects, e.g., refs (16−18). The less well recognized feature is that polydispersity makes PE–S systems multicomponent systems.^19^ Thus, the *molar* surface excess and bulk concentration of a high MW polymer species will generally be low and its Gibbs behavior will be different from that of a low MW species. The few attempts to observe individual components directly have not been successful, e.g., ref (20) although An et al. used NR and surface tension measurements to establish that polydispersity was the probable explanation of anomalous changes in gradient in the Gibbs plot of an aqueous solution of a low MW and relatively monodisperse poly(vinylmethyl)ether.^21^ PE–S mixtures have the extra complexity that PE are also polydisperse in their charge, which means that their surface tension is certain to be time dependent, whether dilute or concentrated. In typical dilute PE–S solutions, an approximate steady state in a PE–S system generally occurs on the scale of an hour or two, but systematic measurements over longer periods show that parts of the surface tension range may change over different periods of 10 or more hours.^10^ Notwithstanding this general non-equilibrium of PE–S systems, a better understanding of the application of the Gibbs equation at low concentrations, where the roles of both surface and bulk behavior should be more clear, is a necessary starting point for understanding the average surface behavior at higher concentrations. Although the low concentration *bulk* behavior of PE–S systems has been quantitatively characterized, e.g., refs (22,23), a significant body of interpretation of the surface behavior at higher concentrations makes little reference to this better understood low concentration range, e.g., refs (18,24−26). In this paper we use the Gibbs and Butler equations to refine our earlier attempts to analyze the surface behavior of strong PE–S complexes at low concentrations^27−30^ and to extend it to weak PE–S systems. Weak PE systems are often branched but here we restrict the analysis to linear PE–S systems. The data used are all in the literature.

### Surface and Solution Behavior of Dilute PE–S Mixtures

The requirements for a quantitative analysis of the surface behavior of PE–S mixtures can be assessed by comparison with studies of mixtures of surfactants above the critical micelle concentration (CMC). Equilibration is relatively easy in micellar mixtures and the analysis of experimental mixed adsorption data has reached a level where non-ideal mixing in both micelles and at the A–W surface can be fully characterized above and below the CMC using the Gibbs and Butler equations with independent measurements of the compositions of surface, solution and micelles.^13,31^ The bulk properties of an aqueous solution of two surfactants below the mixed CMC are little affected by interactions between the surfactants. However, at an interface, the adsorbed molecules are close enough to interact significantly, e.g., an attractive interaction between the surfactants at the A–W surface will lower the surface tension relative to that of a non-interacting mixture and will also cause the surface composition to be closer to equimolarity than the overall composition. The surface composition is therefore generally different from that of the bulk solution and such differences have been difficult to explore experimentally.^32^ The situation is further complicated if there is aggregation in the solution. Thus, even if the surfactants mix ideally in the mixed micelle, the micellar composition is usually different from that of the free surfactants in the surrounding solution.^33,34^ The optimum geometrical arrangement of surfactants in micelles also differs from that at the flat A–W surface, which means that the interactions in the micelles affect the CMC and the micellar composition, and may also cause differences in the bulk monomer composition from those below the CMC. All these features affect the surface tension and the characterization of the A–W surface then requires measurements of the surface tension, the surface excess of each component, and the fractions of each monomer both in micelles and in the free solution. The ways these interactions combine quantitatively in binary and ternary surfactant mixtures,^35^ and even of a quinary mixture,^36^ have been fully demonstrated by a combination of surface tension, NR and small angle neutron scattering measurements.^13^ By analogy with binary micellar systems, the minimum starting information for understanding the equilibrium surface properties of a PE–S system must include (i) the surface tension behavior, (ii) the composition of the layer adsorbed at the A–W surface, (iii) the compositions of any different equilibrium complexes, soluble or insoluble, and (iv) the solute compositions/activities.

The composition of a two solute layer at the A–W interface can be determined directly by measuring the NR from the mixed surface using null reflecting water as solvent with separate measurements for each deuterated component, i.e., isotope effects are neglected. However, for aqueous PE–S systems it is more convenient to measure the surface of the deuterated surfactant with the non-deuterated polymer in NRW and then the same deuterated surfactant–non-deuterated polymer mixture in D_2_O. In the first case the polymer contributes negligibly to the reflectivity but in the second case the reflectivity of the surface of the D_2_O solution of deuterated surfactant is altered by the presence of approximately null scattering polymer in the surface layer.^37^ Making the assumption that the space of the layer is fully occupied by surfactant, polymer and water, the volume occupied by the polymer can be calculated from the two measurements. The number of polymer segments adsorbed per surfactant can then be calculated using the known segmental volume. The accuracy of this method is not as high as measuring the deuterated species separately, but it is reliable enough to demonstrate deviations from the commonly assumed surface composition of 1:1 surfactant:charged segment and it can be applied over a wider range. The characterization of the behavior in the bulk solution is more difficult, especially in the range in which the total surfactant concentration, *s*_total_, is comparable with that of the polymer segments.

The pattern of binding in bulk PE–S solutions is well established when *s*_total_ is less than the number of polymer segments, mainly by means of surfactant electrodes, which measure the activity of the free surfactant in a PE–S mixture as surfactant is added to a fixed concentration of PE.^22,23^ The fractional binding isotherm of surfactant to polymer is determined by equating this activity to the *free* surfactant monomer concentration, *s*_free_, and combining it with the known value of *s*_total_ to determine the bound fraction. Such measurements have established that critical aggregation (CAC) on the polymer chain starts at a low surfactant concentration and is driven by cooperative interaction between surfactants with assistance from electrostatic and/or hydrophobic interactions with the polymer chain. The well established Satake–Yang (S–Y) isotherm^22,38^ describes the cooperative binding with just two parameters giving an isotherm that can empirically be divided into three parts.^30^ For a fixed concentration of polymer and a varying concentration of surfactant, cooperative binding forms the extensive middle part, in which there is a strong increase in the fraction, Φ, of surfactant bound to the polymer for only a small change in *s*_free_. Concentrations up to the onset of the CAC define the lower part. Above a limit at Φ_upper_, the binding becomes relatively non-cooperative and more gradual as it becomes more dependent on the interaction of individual surfactant or other ions with vacancies on the already extensively occupied polymer chain. This means that, although *s*_free_ changes only gradually with *s*_total_ below Φ_upper_, it increases more strongly above it.^30^ This pattern is well established in, for example, the measurements of Hayakawa and Kwak for dodecyltrimethylammonium bromide (C_12_TAB)–poly(sodium styrenesulfonate)(PSS)^39^ and of Lee and Moroi for sodium dodecyl sulfate (SDS)–poly(diallyldimethylammonium chloride) (PDDA).^40^ For C_12_TAB–PSS, the non-cooperative binding extends over a change in *s*_free_ about 30× larger than that required for cooperative binding. For SDS–PDDA in 1 mM NaCl, cooperative binding is complete at a bound fraction of about 0.75 and then there is relatively little binding over the next 10-fold increase in *s*_free_. These and similar measurements show that cooperative binding is not maintained up to the formation of a saturated complex, as is sometimes drawn.^18,26^ Since the Gibbs equation connects the surface tension to changes in the activity of the surfactant in the bulk solution, i.e., ≈*s*_free_, the cooperative binding below Φ_upper_ should be associated with a shallow gradient in an experimental surface tension–*s*_total_ plot. However, in the non-cooperative binding range, the surface tension should change strongly with *s*_total_ partly because *s*_free_ now changes more directly with the larger value of *s*_total_ and partly because it starts from a low value.^30^

In the usual experiment, ionic surfactant (SX) is progressively added to a solution of polyelectrolyte (PY)_*N*_. It is convenient to define a surfactant concentration, *s*_*N*_, where the total concentration of ionic surfactant equals the number of polyelectrolyte segments, and to represent the stoichiometry of a PE–S complex as *S*_β_*P*, where in a charge neutral complex the charge is balanced by β being larger or smaller than unity with Y or X counterions (or H^+^ or OH^–^) respectively balancing the charge. Neutralization is dominated by surfactant because of the additional hydrophobic interactions between surfactant chains and/or between surfactant chain and polymer. We refer to this initially formed neutral complex as the near equivalent NE complex. At higher surfactant concentration, a more surfactant rich complex generally forms by binding surfactant aggregates to the NE complex. We refer to this as the aggregate AG complex. When the concentration of added surfactant passes *s*_*N*_, the increasing tendency for surfactants to aggregate results in the formation of the NE complex followed at higher added surfactant by the AG complex. The activity of the NE complex is then the thermodynamic reference point for the surface tension behavior during initial complexation.

**Figure 1:**
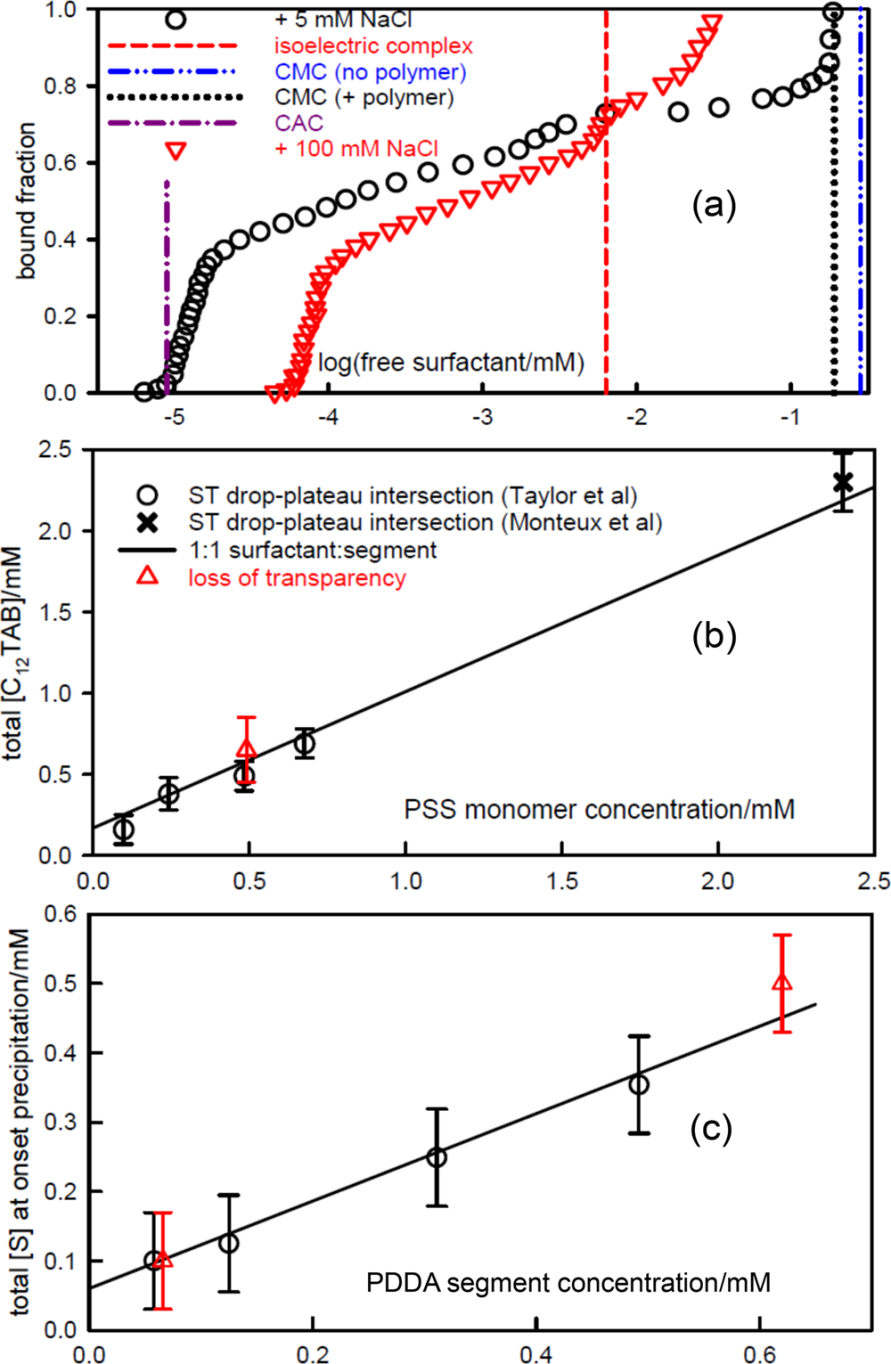
(a) Binding isotherms of C_16_PyCl to PSS at two different NaCl concentrations (PSS segments 1.45 mM), adapted with permission from Ishiguro and Koopal,^41^ copyright 2009 Elsevier. (b) Variation of the onset of the surface tension plateau with *s*_total_ for C_12_TAB–PSS (see also Figure 4) with no added electrolyte using data from Taylor et al.^8^ and Monteux et al.,^42^ and the onset of cloudiness from data of Varga and Campbell.^43^ (c) Variation of the onset of precipitation with *s*_total_ in SDS–PDDA mixtures +100 mM NaCl at different polymer concentrations. The circles in black use data from Staples et al.^6^ and the triangles in red use data from Varga and Campbell.^43^

Because both NE and AG complexes are at or close to neutrality they tend to precipitate, making them difficult to study using surfactant electrodes, which are vulnerable to the attachment of precipitate to the electrode surface. However, the solubility of such complexes varies and it has proved possible to observe their separate formation directly in a group of cationic surfactants attached to PSS. Thus, Ishiguro and Koopal (IK)^41^ used a surfactant electrode to identify two phases in the system alkylpyridinium chloride (C_*n*_PyCl where *n* = 10, 12, or 16)–PSS. Their results for C_16_PyCl at a PSS concentration of 300 ppm (1.45 mM segment concentration) at two background NaCl concentrations (5 and 100 mM) are shown in Figure 1a. The expected CAC is clearly visible at low concentration, and is followed by a range of cooperative binding up to a bound fraction of about 0.4, which in turn is followed by the expected gradual increase in non-cooperative binding up to about 0.7. This range closely parallels those on the related C_12_TAB–PSS system. However, IK’s measurements extend beyond the non-cooperative binding range to reach a plateau corresponding to the formation of a complex at a constant bound fraction. IK attributed this to the formation of a charge neutral complex, whose zero charge they confirmed by separate measurements. The binding plateau for this complex ends with a sharp increase in the bound fraction at a value of *s*_free_ close to the surfactant CMC in the absence of polymer. Thus, IK’s experiment shows a sequence of four distinct stages of aggregation, (i) cooperative binding, (ii) non-cooperative binding, (iii) the formation of a neutral complex, and (iv) a fuller complexation, probably involving micellar aggregates, all of which can be identified clearly in the single binding plot. The initial neutral complex and the fuller complexation correspond with the NE and AG complexes defined above. The larger NaCl concentration also demonstrates clearly the expected opposite effects of an increase in the CAC^44^ but a decrease in the onset of the CMC related AG complexation. The combination of these opposite effects causes the binding plateau of the NE complex to be at its most extended at low added NaCl (5 mM), but it shrinks to a barely visible kink in 100 mM NaCl. IK also found a similar but less clear plateau for the shorter chain C_12_PyCl–PSS system at low NaCl concentrations but no noticeable corresponding effects for C_10_PyCl–PSS.

The fractional binding in the NE complex in Figure 1a is 0.73, i.e., β = 0.73 in the stoichiometric formula *S*_β_*P*. Since the complex was shown to have zero charge, IK suggested that this stoichiometry is a result of incomplete sulfonation and/or partial hydration of the commercial PSS sample. Both of these are known to occur, but at a level that should give a slightly higher charge of 0.80–0.85.^45,46^ The lower fraction of *S* in the measured composition may result partly from this shortfall and partly from a fraction of polymer counterions in the complex. Thus, the NE (near equivalent) complex has zero charge, not all of which necessarily derives from neutralization by the surfactant ion. The key results from IK are that two distinct complexes occur in the vicinity of *s*_*N*_, an NE complex somewhat below segment–surfactant equivalence and an AG complex with an excess of aggregated surfactant, and that it is the NE complex that ends non-cooperative binding. The NE complex therefore largely determines the solution activity of the PE–S complex and hence, via the Gibbs equation, much of the surface tension behavior in the vicinity of *s*_*N*_. A further implication of IK’s results is that the NE complex for C_16_PyCl–PSS remains in liquid form (soluble or as a pseudophase) because the surfactant electrode method would fail if there was precipitation. This conclusion is partly confirmed by Prelesknik et al., who found that the C_16_PyCl–PSS system (with 100 mM NaCl) is soluble up to a bound fraction of 0.7, but then becomes insoluble up to a bound fraction above 1.^47^ Prelesknik et al. suggested that this solubility is a particular feature of the PSS chain, i.e., other cationic surfactant systems using PSS might exhibit a similar solubility pattern. It is then interesting that Varga and Campbell (VC)^43^ have shown, via optical density measurements, that there are two solubility regimes for the system C_12_TAB–PSS (100 ppm or 0.49 mM segments) in the absence of added electrolyte. Precipitation occurs in an *s*_total_ range of 3.5–10 mM but there is a range of cloudiness starting at the lower *s*_total_ of about 0.7 mM, which continues up to the onset of the main precipitation at 3.5 mM. The two concentration ranges are consistent with the general pattern observed by IK if VC’s onset of cloudiness is taken to be the equilibrium onset of NE complexation rather than the formation of kinetically trapped aggregates (as proposed by VC). The Gibbs equation requires that the formation of an NE complex should cause a change from a negative gradient of the surface tension to a near plateau, as observed for C_12_TAB–PSS by Taylor et al.^8^ at four different PE concentrations (shown below in Figure 4), and by Monteux et al.^42^ at a fifth higher concentration. The onset of formation of an NE complex should vary linearly with polymer concentration and occur at a constant fixed value of *s*_free_. This plot is shown in Figure 1b with all six data points from the three independent measurements. Its gradient is close to one (0.85 ± 0.15), as would be expected for equilibrium formation of an NE complex. The higher concentration precipitation observed by VC then corresponds to the onset of formation of the insoluble AG complex. VC did not observe the phase change marked by the change in surface tension at the low concentration end of its plateau (their measurements started above this point). However, more recently, Braun et al.^48^ have verified this sharp change in surface tension for the longer chain C_14_TAB–PSS under conditions where there can be *no* kinetically trapped aggregates.

In the C_12_TAB–PSS system the NE complex is detectable because of its cloudy solution, i.e., it can be thought of as a pseudo–phase, with a close to constant activity. There are also systems where the NE complex is insoluble, which makes it easier to detect. Such precipitation in the SDS–PDDA system with added NaCl (100 mM) has been measured as a function of polymer and surfactant concentration by Staples et al.^6^ and the variation of its onset of with *s*_total_ is plotted in Figure 1c together with two points from a later measurement by Varga and Campbell.^43^ The gradient of this plot gives a stoichiometry of S_0.63_P for the NE complex of SDS–PDDA in 100 mM NaCl in equilibrium with an *s*_free_ of about 0.04 mM (the intercept at a segment concentration of zero). In this case, the surprisingly low stoichiometry may also result from the high concentration of 100 mM NaCl causing more Cl^–^ ions to be incorporated into the NE complex.

An alternative approach for exploring the stoichiometry of the *SP* complex is to prepare it in a pure form and examine its solution behavior directly. This approach has been followed by Piculell et al.,^49−51^ who have prepared exact charge equivalent complexes by direct combination of the pure acid and base forms of surfactant and polyelectrolyte. The majority of these experiments have used the base polyacrylate ion (PAA) with cationic (C_*n*_TA^+^) ions^49^ and the resulting *SP* complexes have been found to be negligibly soluble in pure water. This might suggest that an insoluble NE complex is normally the first complex to be stable over a range of concentration of added surfactant. However, Piculell and co-workers have also shown that the *SP* complex can be significantly solubilized by extra added electrolyte and/or surfactant depending on the initial polymer and the additive.^50,51^ Thus, in the different situation where the NE complex is formed by mixing surfactant with polymers, it is not surprising that the stoichiometry of the NE complex both deviates from *SP* stoichiometry and is not necessarily insoluble, although it may form a distinct phase or pseudophase in pure water.

### The Complexation Region

We divide the surface behavior of dilute PE–S solutions into three concentration ranges of added surfactant, (i) the *complexation* range, extending from low concentration up to the onset of formation of the bulk NE complex, (ii) the *intermediate* range extending to the approximate onset of formation of the bulk AG complex, and (iii) the *surfactant excess* range. This division into three concentration ranges is simpler than that used in our previous paper^30^ and has a clearer thermodynamic basis. Thus, the surface behavior in the complexation range can be explained in terms of the activity of the PE–S complex and hence is determined approximately by the Gibbs equation. However, in the *intermediate* range the surface tension will depend on a combination of both the PE–S complex and the excess free surfactant that starts to be present at the higher concentration. This has to be described in terms of the Butler equation, which requires that the partial surface pressures of each of the complex and the free surfactant are equal at equilibrium.^52,53^ The *surfactant excess* range must also be described by the Butler equation but now the species competing for the surface are mainly the free surfactant and the surface form of the bulk AG complex.

A simple starting point for the surface tension behavior of the complexation region is then to consider the adsorption of a polyelectrolyte PX_*n*_ on its own, i.e., to apply just the Gibbs equation for a single component. This can be written in terms of the adsorption and bulk activity of either the neutral species, P_*n*_X_*n*_, or of its dissociated components (P_*n*_^*n*+^ + *n*X^–^),^14,54^ i.e., either
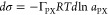
1where σ is the surface tension, Γ is the surface excess and *a*_PX_ is the activity of the undissociated species, or
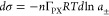
2where
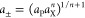
3and *a*_±_ is the mean activity of the dissociated adsorbed species, with similar expressions holding for the mean concentration, *c*_±_, and mean activity coefficient, *f*_±_. In the experimental situation where the polymer concentration is held constant while the counterion (X) concentration is varied, the surface tension variation is then
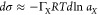
4

Strong PEs are generally not very surface active but if surfactant ions of charge opposite to that of the polyion are added to a PE solution, the surfactant might be expected to replace the majority of the counterions to generate a surface active complex and PE–S complexes in the bulk solution. If the surfactant ion replaces the normal counterion *completely* so that it is (*SP*)_*N*_ that is adsorbed, then the Gibbs equation will have the same form as eq 4 but with surfactant replacing counterion. If the activity of the surfactant is dominated by its strong binding to the polymer, the concentration of free surfactant, *s*_free_, becomes a reasonable approximation for its activity, and eq 2 can then be written as
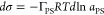
5

This is similar to the equation used by Buckingham et al.^14^ in a study of SDS mixed with poly(l-lysine). The assumptions of eq 5 then become (i) all the counterions involved in both the bulk and surface complexes are surfactant ions and (ii) the bulk and surface complex have the same *SP* stoichiometry. The key feature of eq 5 is that, for a fixed total PE concentration, the surface tension depends directly on *s*_free_, which is known from surfactant electrode measurements, and the surface excess, Γ_S_, which can be determined by NR. A fuller discussion has been given by Warszynski et al.^55^

There are two complications. The first is that in a weak or incompletely charged polymer, there will be two types of complexation corresponding to different segment–ion pairs, driven by one or other of eqs 2 or 5, which then become intractable to handle, especially if the un-ionized sets of segments are significantly surface active, which is expected, for example, for C_12_TAB-poly(acrylate)(PAA). Comparable situations, may arise for (i) chain branching, which leads to different binding sites, (ii) partially charged polyelectrolytes,^56^ and (iii) many commercial polyelectrolytes, e.g., PSS, which is typically only 80% charged. In such cases, eq 5 only applies to changes in the surfactant concentration but the limiting properties and the intrinsic surface activity may be significantly different from the fully charged species. The second complication is that if the total electrolyte in the system is below a certain threshold and if the spacing of charges on the polymer is below the Bjerrum length, *L*_*b*_ (≈7 Å), counterion condensation^57−60^ may occur with the result that the counterion activity is constant and the activity of the polymer becomes that of the undissociated PX_*n*_, i.e., the appropriate form of the Gibbs equation becomes eq 1. If the activity of the surfactant is again assumed to be dominated by its strong binding to the polymer, surfactant replaces counterion in eq 1 to give the alternative equation
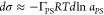
6

If the surfactant ion becomes part of the counterion condensation its activity will be constant over a range of low concentration and, at a constant polymer concentration, eq 6 then requires there to be little change in surface tension as surfactant is added. This effect will only be observed if (i) the PE has a charge separation lower than *L*_*b*_(61) and (ii) the added electrolyte is lower than a particular threshold level. There are also uncertainties about the composition of the counterion condensate and its evolution as surfactant is added.^62^ In the following we therefore examine complexation with and without electrolyte separately.

### The Complexation Region without Added Electrolyte

**Figure 2:**
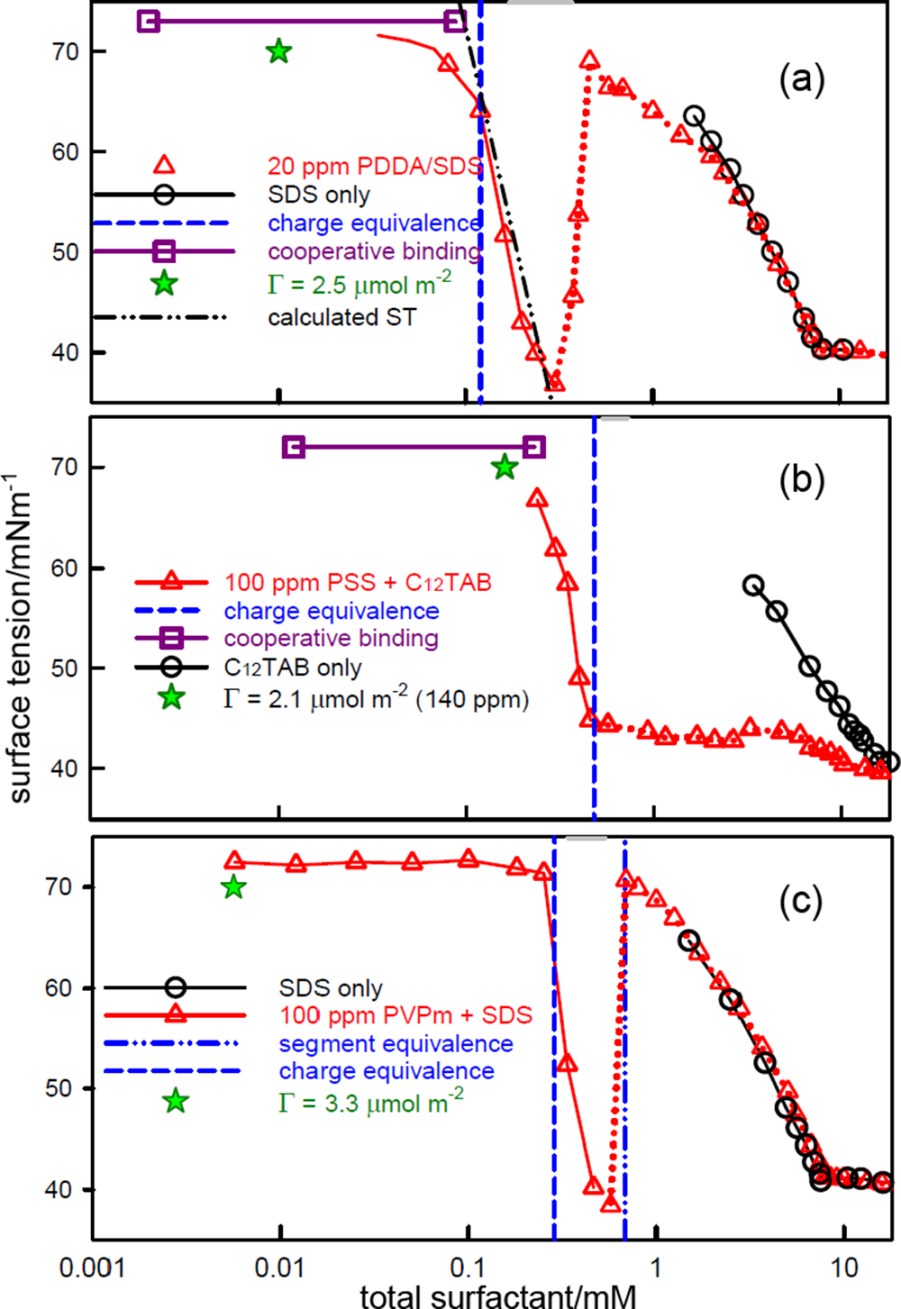
Surface tension of (a) SDS–PDDA (20 ppm), (b) C_12_TAB–PSS (100 ppm), and (c) SDS–PVPm (100 ppm), as a function of *s*_total_. Continuous lines mark the complexation region and dotted lines mark the remaining regions. Vertical dashed lines mark nominal charge equivalence (the dot–dash blue line for SDS–PVPm marks segment equivalence). Large square points and horizontal lines mark cooperative binding for PDDA–SDS^40^ and PSS–C_12_TAB.^39,62^ Green stars mark the surface excess of surfactant at the corresponding polymer segment and surfactant concentration, except for C_12_TAB–PSS, where it refers to the higher concentration of 140 ppm PSS. The dash–dot black line in (a) is the surface tension calculated using eq 9 as described in the text. The data are redrawn from Penfold et al.^63^ and Taylor et al.^8,64^

**Figure 3:**
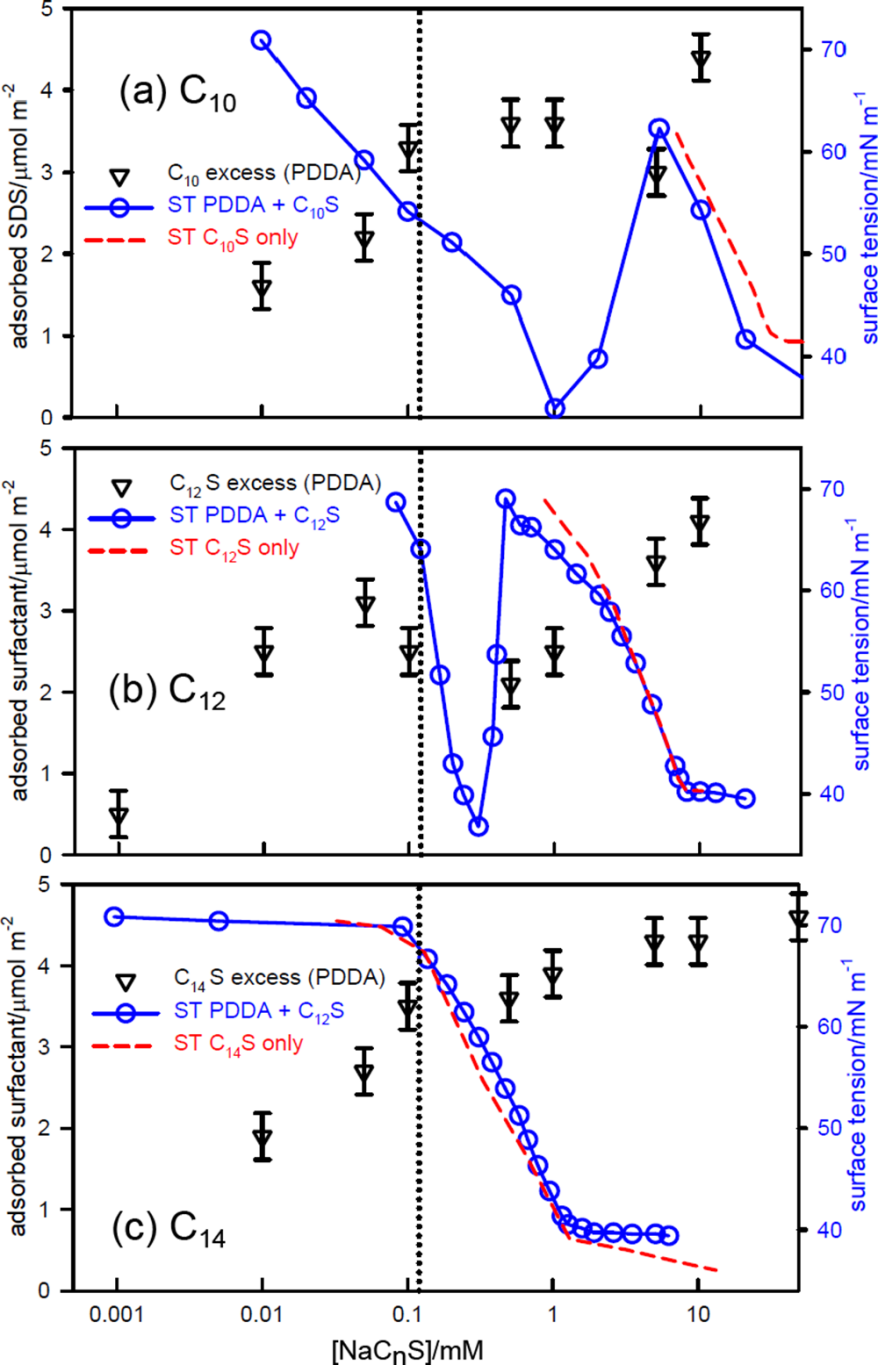
Surface tension of SC_*n*_S–PDDA (20 ppm) mixtures with *n* equal to (a) 10, (b) 12, and (c) 14, and no added electrolyte. The surface excesses are marked as points and the dotted lines mark *s*_*N*_. The data are from Penfold et al.^63^

Figure 2 shows the surface tension variation with log(*s*_total_) for three PE—S systems, SDS–PDDA, C_12_TAB–PSS and SDS-PVPm (poly(vinyl-2-pyridinium chloride)), all without added electrolyte, together with the known behavior of the free surfactants. PVPm is a weak PE but PDDA and PSS are strong polyelectrolytes and they are expected to ionize completely in the absence of surfactant. Four obvious features of the surface tension are (i) the high and approximately constant initial value at low concentration, (ii) the onset of the initial drop at an *s*_total_ that is slightly below *s*_*N*_, (iii) the large negative gradient of the main drop, which is greater than for each surfactant on its own near its respective CMC, and (iv) that the drops in surface tension for SDS–PDDA and SDS–PVPm reverse sharply at a concentration approximately between the complexation and intermediate regions, whereas the surface tension simply falls to an approximate plateau for C_12_TAB–PSS. The onset of the drop in surface tension is proportional to the polymer concentration, as shown for C_12_TAB–PSS in Figure 3.

The high initial surface tension in all three examples shown in Figure 2 is accompanied by significant surfactant adsorption, measured directly by NR, examples of which are the points and values marked in the figure by green stars. If surfactant is present at the A–W interface with *no* accompanying drop in surface tension, the Gibbs equation requires that the mean activity of the adsorbate in the bulk solution must be *invariant* with concentration. Counterion condensation is expected to occur for PEs where the separation of the ionized units is less than the Bjerrum length, *L*_B_ (about 7 Å in water), but it is disrupted by added electrolyte. It is therefore not expected to occur in surface electrode measurements, where electrolyte is added to ensure the correct functioning of the electrode. However, it may occur during the initial addition of surfactant in a surface experiment if a fraction of surfactant ions is incorporated into the counterion condensate.^62^ Counterion condensation might then persist up to the CAC. If this occurs, the mean activity of the surface active species in solution will remain at a low level until cooperative binding is complete. In such a situation the surface tension will remain approximately that of water up to the completion of cooperative binding, even though there may be an almost complete monolayer of complex adsorbed at the surface. The surface tension is high because, when the surface area is increased, the entropy penalty associated with replacing a complex of low bulk activity is high, even when there is a favorable mechanism selectively stabilizing the surface layer.^65^ For all three systems shown in Figure 2 the high initial surface tension plateau with a significant level of adsorbed PE–S complex means either that there is counterion condensation closely followed by cooperative binding in all three systems or that none of them is equilibrated (discussed separately in a later section).

The high surface tension at low concentration can be disrupted by varying the chain length of the surfactant because this alters the balance of counterion condensation and complexation. Reduction of the chain length of the surfactant reduces the cooperativity in the binding, which means that the mean activity of the adsorbed species increases more strongly with concentration. This increases the gap between the concentration at which surfactant disrupts counterion condensation and that of the onset of cooperative binding, and causes the mean activity to increase more rapidly above this onset. This effect is seen in the effect of chain length on the surface tension behavior of SC_*n*_S–PDDA systems with *n* equal to 10, 12, and 14 carbon atoms, shown in Figure 3. Equivalence, (*s*_*N*_), marked by a dotted line, is the same for all three systems but the CMC, marked by a dashed red line, decreases substantially from C_10_ to C_14_. The SC_12_S system has the initial surface tension plateau expected when counterion condensation merges into cooperative binding, and this ends in the expected steep drop in the vicinity of *s*_*N*_, which we discuss further below. The binding of SC_10_S to PE is, however, much less cooperative with the result that the surface tension decreases with concentration more like a simple surfactant solution, although at a much lower surfactant concentration, with the onset of the decrease disrupting the initial counterion condensation. Cooperative binding in the SC_14_S system is expected to be stronger than for SC_12_S (SDS) and there is therefore an initial plateau. However, the surface activity of free SC_14_S is now that much higher that the free surfactant becomes the dominant surface active species before complexation is complete. Its gradient is less steep than for SC_12_S, as we discuss in a later section, but it is significantly steeper than that of SC_10_S, again confirming the low cooperativity of complex formation in the latter. Counterion condensation can also occur in some weak PEs, and the PVPm shown in Figure 2c is an example. Thus, a more recent study of the state of ionization of PVPm shows that, under the conditions of the NR experiment, which used solutions of fully quaternized PVPm, the PVPm would have been about 45% ionized.^66^ The mean separation of the segments in PVPm is about 2.8 Å and the charge separation therefore drops below *L*_*B*_ when ionization is more than about 40%, i.e., the conditions of the SDS–PVPm system used in Figure 2c would also result in counterion condensation at low concentrations, even though the PVPm is nominally less than half ionized.

**Figure 4:**
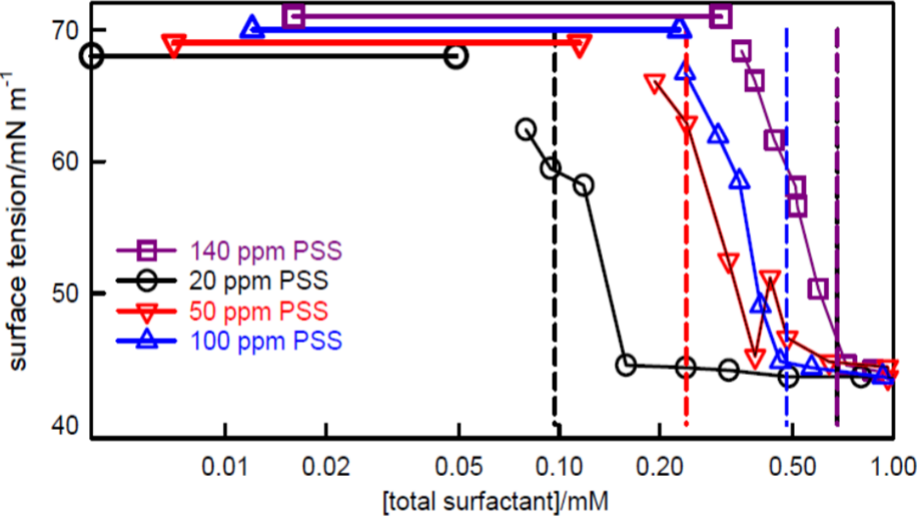
Surface tension of C_12_TAB–PSS mixtures at different polymer concentrations. The large symbols connected by horizontal lines mark the concentration ranges of cooperative binding using binding data from Hansson and Almgren^62^ and the assumption of a limiting cooperative binding fraction of 0.45, an approximate estimate based on measurements in the presence of electrolyte.^39^ The dashed lines mark *s*_*N*_, the nominal charge equivalence. The surface tension data is from Taylor et al.^8^

Surfactant binding isotherms have been fully characterized for both C_12_TAB–PSS^39,62^ and SDS–PDDA.^40,67^ During cooperative binding the bound fraction increases substantially while *s*_free_, changes relatively little, i.e., most of the surfactant added in this range becomes part of the complex. Thus, *s*_total_ increases substantially while *s*_free_, which is approximately the activity of the surfactant ion, changes by only a small amount, with the result that there is little variation of the surface tension with *s*_total_.^30^ A high level of adsorption is therefore maintained at a low *s*_free_ up to the completion of cooperative binding at Φ_upper_ × *s*_*N*_ (both quantities as defined in the previous section). Above this concentration, cooperative binding ends and *s*_free_ converges sharply toward *s*_total_, i.e., the increase in *s*_free_ and the corresponding decrease in surface tension both become large with respect to *s*_total_. The onset of the sharp negative surface tension gradient is therefore determined by the value of the maximum cooperatively bound fraction, Φ_upper_. Making the approximation that *s*_free_ is constant while the binding remains cooperative, and using the experimentally known values of the onset of binding and the cooperative binding range, leads to the approximate ranges marked as horizontal lines in Figure 2a,b and at different PSS concentrations in Figure 4. Since Φ_upper_, is significantly less than unity (the approximate values of Φ_upper_ are 0.45 and 0.7 for C_12_TAB–PSS and SDS–PDDA, respectively) and, since this point is also the start of the initial drop in surface tension, this drop occurs *below s*_*N*_. This is particularly clear for C_12_TAB–PSS at lower concentrations of polymer (Figure 4).

The third feature of the surface tension curves is the unusually steep gradient of the main fall in tension. The cause of this fall is as already described, i.e., immediately following completion of cooperative binding, *s*_free_ changes more steeply than *s*_total_.^30^ The effect can be calculated from estimates of *s*_free_ at the completion of cooperative binding and at the onset of the low surface tension plateau. These have been directly measured for SDS–PDDA using 100 ppm PDDA (0.62 mM polymer segments) in 1 mM NaCl by Lee and Moroi (LM)^40^ and later by Nizri et al.^67^ LM’s data at 298 K give a well-defined value of Φ_upper_ of about 0.7 and the cooperativity is low above this point, i.e., the binding fraction changes only gradually with concentration. The value of *s*_free_ can be estimated to be about 0.002 mM at the completion of cooperative binding and its value scales with the total polymer concentration, so that the equivalent concentration for the 20 ppm data of Figure 2a would be about 0.0004 mM. The maximum value of *s*_free_ at the end of the drop in surface tension can be calculated from *s*_total_ less the amount of cooperatively bound surfactant. These two values combined with the known value of Φ_upper_ = 0.7 at the original polymer segment concentration (0.124 mM), give a maximum value of *s*_free_ of 0.21 mM at the lowest tension. Application of the Gibbs equation using these values and eq 5 with the directly measured surface excess of 2.5 μmol m^–1^ gives a drop in surface tension of 39 mN m^–1^ shown as a double dot–dashed line in Figure 2a. This is a much larger drop than the corresponding value using the change in *s*_total_, which would only be 8 mN m^–1^. While there are several approximations in this estimate, the steep drop in surface tension at the switch from cooperative to non-cooperative binding, which is a common feature of its behavior in many PE–S systems, is accounted for by the Gibbs equation. A similar analysis, also based on independent experimental measurements of the binding isotherms, accounts for the sharp decrease in surface tension in the C_12_TAB–PSS system.

The fourth feature is more complicated in that it depends on the bulk phase behavior and the compositions of the surface and bulk complexes. In the simple analysis of the Gibbs equation given above, where the complex is assumed to have *SP* stoichiometry in both bulk solution and in the adsorbed layer, the surface tension should reach a low constant value when the bulk NE complex forms, which may not have exact *SP* stoichiometry. However, the more important result is that the NE complex must persist over a range of further addition of surfactant and should therefore result in a low plateau in the tension at this point, as observed in the case of C_12_TAB–PSS (Figure 4). The concentration at which this plateau starts would then be the onset of the formation of the NE complex and should therefore be proportional to the amount of polymer, which is clearly seen in Figure 4 and in the plot of Figure 1b. There is no precipitation at this point in the C_12_TAB–PSS system, demonstrating that the NE complex formation is not necessarily a precipitate.

The SDS–PVPm system has been included in this section because it behaves similarly to the strong SDS–PDDA system. In particular, it has a similar high initial surface tension plateau, which we attribute to the combination of counterion condensation and strong cooperativity. In the conditions of Figure 2c, where the total segment concentration for PVPm is 0.7 mM, approximately 0.3 mM should be be ionized,^66^ which is sufficient to cause counterion condensation. The line denoting charge equivalence and the surface tension pattern as a whole is remarkably similar to that of SDS–PDDA, apart from the relative position of *s*_*N*_. The composition of the surface was not measured in the absence of added electrolyte, but the measured surface excess of SDS in the range of the high surface tension plateau is higher than for either of the two strong polyelectrolytes in Figure 2a,b. Although the stoichiometry was not measured in the absence of electrolyte, it was found to be close to *SP* in electrolyte. We discuss this system in more detail in the section on weak PE–S systems.

### The Complexation Region with Added Electrolyte

**Figure 5:**
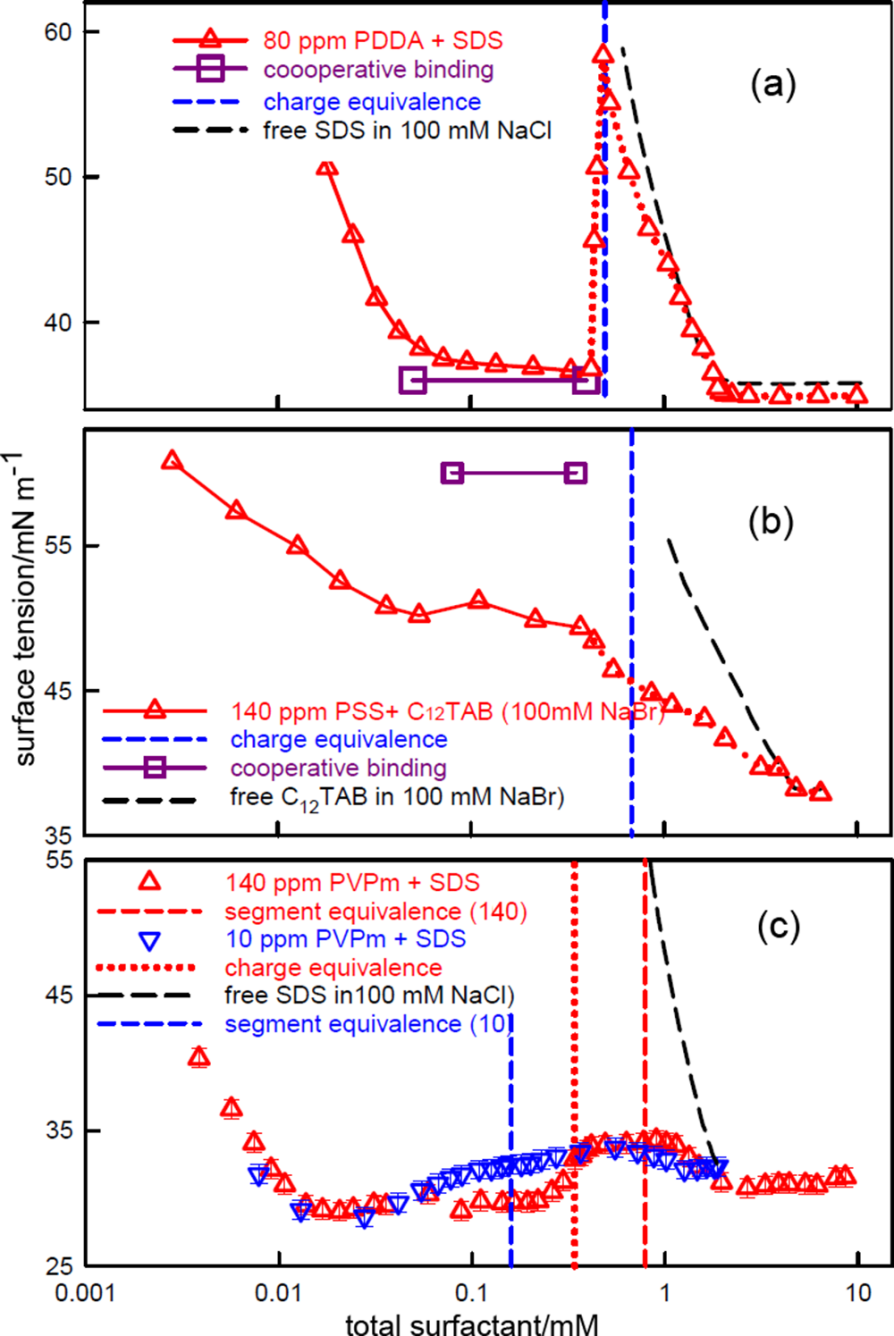
Surface tension of (a) SDS–PDDA (80 ppm), (b) C_12_TAB–PSS (140 ppm), and (c) SDS–PVPm (140 ppm), all with 100 mM NaCl or NaBr, redrawn from Staples et al.^6^ and Taylor et al.^64^ and each plotted against *s*_total_. Complexation is marked by a continuous line and the remaining region by a dotted line. Blue dashed lines mark nominal charge equivalence in (a,b) and for the 10 ppm sample in (c). The large square points and the horizontal lines in (a,b) mark cooperative binding measured for SDS–PDDA^67^ and C_12_TAB–^39,62^ in 100 mM NaCl/NaBr.

Figure 5 shows the surface tension behavior of the same three PE–S systems but now in 100 mM NaCl (or NaBr in the case of C_12_TAB–PSS). The concentration scale is the same as in Figure 2, but the polymer concentrations are different. The complexation and remaining regions are again distinguished by continuous and dotted lines, respectively. The obvious difference between Figures 5 and 2 is that there is no initial high surface tension plateau. Counterion condensation is suppressed by the addition of electrolyte and the surface tension then varies more like that of a surfactant on its own, but at much lower concentration. Electrolyte also increases the CAC by about an order of magnitude.^44^ The bulk binding isotherms in added electrolyte have been measured for SDS–PDDA and C_12_TAB–PSS using surfactant electrodes^39,67^ and their known ranges of cooperative binding are shown as horizontal lines in Figure 5a,b. As in the absence of electrolyte, *s*_free_ becomes approximately constant at the onset of cooperative binding, but now this occurs at a low surface tension.

For the two strong polyelectrolyte systems in the complexation region, the behavior of the surface tension, the surface coverage, and the complexation in the bulk solution are consistent with the approximate Gibbs equation (eq 5), indicating that these systems are equilibrated in the ranges marked by continuous lines in Figures 2 and 5. Similar surface tension behavior has also been observed in a range of C_*n*_TAB-PSS with varying *n*,^68^ including C_12_TAB–PSS, by Monteux et al.,^42^ Noskov et al.,^69^ and recently C_14_TAB–PSS by Braun et al.^48^ both with and without added electrolyte, which, within the complexation region, show similar behavior to that obtained by Taylor et al.,^68^ i.e., a high plateau below charge equivalence in the absence of electrolyte with a fairly sharp drop in surface tension in the vicinity of charge equivalence. The sharp peak in the tension of SDS–PDDA has been separately confirmed by Varga and Campbell.^43^

There are again differences in the behavior of SDS–PVPm. First, although the addition of electrolyte normally causes a significant increase in the onset of cooperative binding, as observed for the two strong PEs, the effect is substantially weaker for SDS–PVPm. Thus, the onset of cooperative binding (the onset of the surface tension plateau) starts at a concentration not very different from that in the absence of electrolyte. The addition of electrolyte increases the ionization of free PVPm by approximately 15%^66^ and this evidently increases the strength of the cooperative binding by about as much as the addition of electrolyte weakens it. The effect of this added stabilization of cooperativity results in an unusually large range of concentration between the onset of cooperative binding and the final charge neutralization.

### Surface Stoichiometry

The derivation of the approximate surface tension, eq 5, assumes that the *SP* complex is the only adsorbing species. There are three situations where this assumption is likely to break down, (i) when the stoichiometry of the bulk complex is different from *SP* because this causes the bulk activities of the two complexes in solution to vary differently with surfactant concentration, (ii) when complexation is largely complete and *s*_free_ increases to a level at which free surfactant competes with the PE–S complex for the surface, and (iii) when PE–S complex and un-ionized weak PE–H (or OH) complex compete for the surface. In (ii) and (iii) the Butler equation must be used. However, NR experiments show that the stoichiometry of the surface complex is often both different from *SP* and is maintained over a wide range of concentration. Thus, in the complexation region the measured (NR) surface stoichiometries of SDS–PDDA (in 100 mM NaCl) and C_12_TAB–PSS (with and without added electrolyte) are approximately S_0.5_P and S_2_P^6,8^, respectively. The relative constancy of these stoichiometries over the dilute range of added surfactant indicates that they are particularly favored and thus dominate adsorption during complexation. Since adsorption at the A–W surface must be neutral overall, the surface composition for SDS–PDDA is then expected to adjust to approximately *S*_1/2_*Y*_1/2_P where *Y* is the polymer counterion. Following the earlier derivation, the approximate Gibbs equation can then be written as
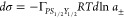
7where *a*_±_ is the mean activity of the hypothetical equivalent bulk complex, i.e.,
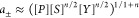
8

The surface tension and surface excesses are respectively denoted by σ and Γ, as before. Since the concentrations of polymer and counterion are constant in the surface tension measurements, eq 8 reduces to
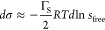
9

Thus, the general contribution of this stoichiometry to the gradient of the surface tension plot should be approximately to halve it.

The stoichiometry of the surface complex of C_12_TAB–PSS is approximately S_2_P.^8,64^ Neutrality at the A–W interface then requires that the complex contains an extra surfactant counterion. The approximate Gibbs equation then becomes
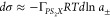
10where *a*_±_ is the mean activity of the hypothetical bulk complex, PS_2_X, i.e.,
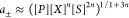
11

This is more difficult to reduce than for SDS–PDDA in that [X] ≈ *s*_total_. However, in the absence of added electrolyte, [X] may contribute to an increase in the magnitude of the surface tension gradient and hence to the unusually steep variation in the surface tension observed as charge neutralization is approached (see Figures 2 and 4). The presence of electrolyte and the effect of the relatively large and now *constant* value of [X] may be factors causing the noticeably shallow slope in the tension observed in the low concentration range of Figure 5b.

### The Intermediate Region and the Stoichiometry of Adsorption

The intermediate region for SDS–PDDA (dotted lines in Figures 2 and 5) starts with an unusual *positive* gradient in the surface tension–*s*_total_ plot, whether or not electrolyte is present. Thermodynamically, this can only occur if the activity of the adsorbed species decreases on adding surfactant. With SDS–PDDA, the NE complex precipitates at the start of the intermediate region. If the surface complex (SC) and NE complex have the same stoichiometry, the mean activity of the hypothetical SC complex in solution then remains approximately the same as that of the bulk NE complex as further surfactant is added. This would cause the surface tension–*s*_total_ plot to have a plateau starting from the onset of NE formation. However, the SC stoichiometries are *S*_0.5_*P* and *S*_2_*P* for SDS–PDDA and C_12_TAB–PSS respectively, and the activities of the SC and NE species therefore vary differently with added surfactant above the onset of NE complex formation.

The effects of this difference can be approximated by assuming initially that the NE phase has *SP* stoichiometry. The initial low surface tension limit is then reached at the onset of formation of the NE complex, which occurs at charge equivalence plus a small but well-defined amount of free surfactant, (*s*_*N*_ + δ), as shown in the two extrapolations of Figure 1b,c. The simplest case is when the SC and NE complexes have approximately the same *SP* stoichiometry. The formation of the NE complex is then complete at a total surfactant concentration of (*s*_*N*_ + δ) and further addition of surfactant becomes free excess surfactant in the solution. The concentration of this excess is usually well below the CMC of surfactant on its own and it therefore does not contribute significantly to the surface tension. In this situation, precipitation or pseudophase separation of the NE complex therefore results in an approximate surface tension plateau, approximate because if the NE complex forms a pseudophase there will be some limited variation of its activity with concentration. When the NE complex has *SP* stoichiometry but the SC complex contains a higher fraction of surfactant, i.e., *S*_θ_*P* with θ > 1, then the mean activity of the SC complex above the onset of formation of the NE complex at (*s*_*N*_ + δ) can be determined from the mean of the activity of the surfactant that is part of the NE species and that of the excess free surfactant. Since the former is constant and more dominant (see eq 3) the mean activity of the hypothetical SC complex in the bulk solution will increase with *s*_total_ more gradually than below the onset of NE formation. Hence there will be a low negative gradient in the surface tension above this onset, as observed in C_12_TAB–PSS (see Figures 2b and 4).

The activity behavior is more complicated when the stoichiometry of the SC complex is *S*_θ_*P* with θ < 1. For simplicity, we again assume that the NE complex has *SP* stoichiometry and that its precipitation again occurs at (*s*_*N*_ + δ). The activity of the SC complex in the bulk solution now reaches a high enough value for SC to adsorb significantly at concentrations below (*s*_*N*_ + δ), mainly because of the extensive cooperative binding that occurs at lower concentrations. However, at some point the lower stoichiometry surface species will start to change to the higher stoichiometry, non-surface active NE complex. In the absence of added electrolyte, the formation of the NE complex may then result in the normal initial drop in the surface tension being interrupted and reversed in its activity before it has been completed. The extent and position of this reversal will depend on quantitative details of the cooperativity, which are not available. However, the concentration of the point of reversal should occur at a surfactant concentration approximately proportional to the total amount of PE in the solution. The reversal in surface tension is seen for SDS–PDDA and SDS–PVPm in Figure 2a,c.

**Figure 6:**
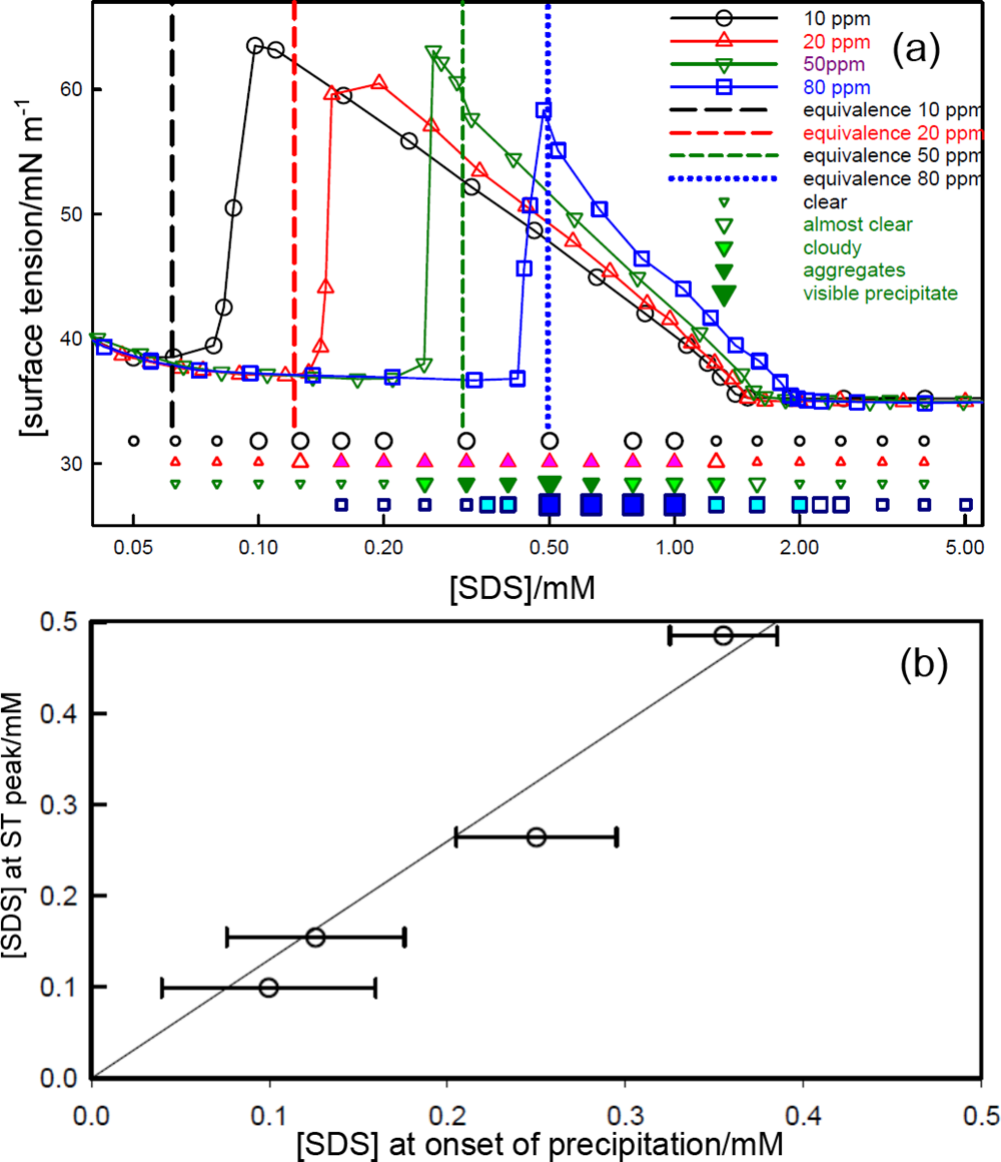
(a) The surface tension and precipitation behavior of SDS–PDDA at different PDDA concentrations in 100 mM NaCl. The extent of precipitation and the surface tension are marked with matching symbols with the size of the former symbols following the sequence small unfilled, large unfilled, large pale filled, large dark filled, extra large filled. Vertical lines indicate the total polymer segment concentration (data from Staples et al.^6^). (b) Plot of *s*_total_ at the tension peak against the surfactant concentration at the onset of precipitation, using the data in (a). The line is calculated from the ratio of the stoichiometries of the NE and SC complexes.

The effect of the low surfactant stoichiometry of the SC complex is clearer in added electrolyte because this causes the system to reach a low surface tension plateau at the *onset* rather than the *completion* of cooperative binding. This plateau is maintained up to the formation of the NE complex, which is a precipitate in the case of SDS–PDDA. The onset of this precipitation, the surface tension, and the state of precipitation for four different PE concentrations are shown in Figure 6a. These onsets have already been used in Figure 1c to estimate the stoichiometry of the NE complex as *S*_0.63_*P*, slightly higher than the *S*_0.5_*P* stoichiometry of the SC complex measured directly using NR. Thus, the formation of the NE complex progressively reduces the solution activity of the SC species, just as in the absence of added electrolyte. The surface tension remains on a plateau only while the mean activity of the SC complex remains above a threshold level, but when it drops below this level the tension starts to increase. The surface tension peak then marks the point at which the activity drops to close to zero. Figure 6b plots the measured *s*_total_ at the peak as a function of *s*_total_ at the onset of precipitation for the polymer concentrations shown in Figure 6a. Since the concentration of the surface active species at the peak should be close to zero, the gradient of this plot is the ratio of the surfactant stoichiometries for the NE and SC complexes (0.63/0.5) drawn as the straight line. The horizontal error bars reflect the combined uncertainty in the measurements of the concentration at the surface tension peak and the onset of precipitation.

**Figure 7:**
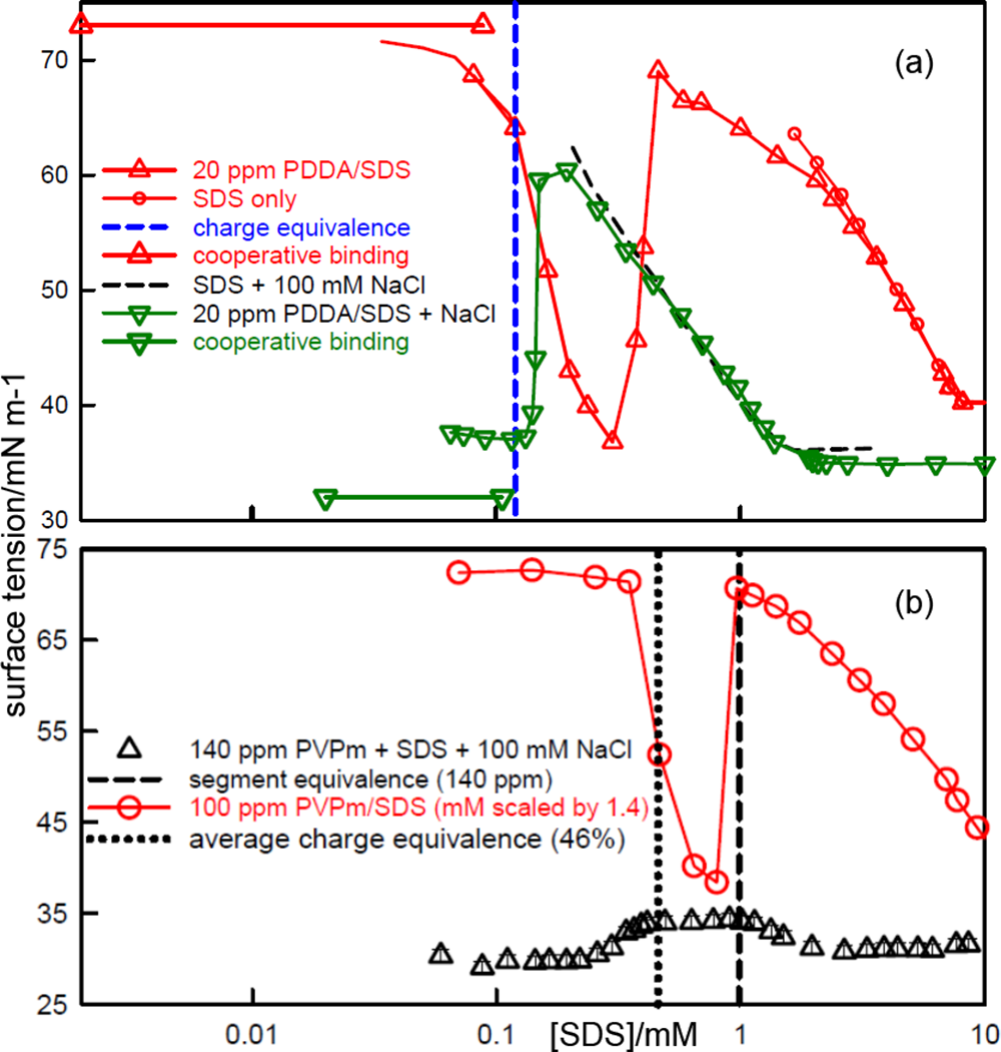
Comparison of the surface tension behavior of (a) SDS–PDDA solutions at a PDDA concentration of 20 ppm with and without 100 mM NaCl (data redrawn from Staples et al.^6^ and Penfold et al.^63^) and (b) SDS–PVPm solutions with and without NaCl. In (b) the solution with electrolyte is at a PVPm concentration of 140 ppm. The solution without electrolyte was measured at a PVPm concentration of 100 ppm and its concentration has been scaled by 1.4 to allow a more direct comparison. The data are redrawn from Taylor et al.^64^

The effect of electrolyte on the increase of surface tension with concentration is examined more closely for 20 ppm PDDA in Figure 7a. The high [NaCl] is expected to result in chloride ions displacing a fraction of negative surfactant ions from both SC and NE complexes. Given that the hydrophobic effect is particularly strong at the A–W interface, the displacement by chloride ions might be expected to be stronger for the NE complex than for the SC complex. Addition of NaCl would then both lower the onset of formation/precipitation of the NE complex and reduce the gap between this onset and depletion of the SC complex. Since the extent of depletion should be reduced if it occurs over a shorter concentration range, the increase in surface tension at the anomaly should also be reduced. All three effects are clear for SDS–PDDA in Figure 7a. In addition, there is no observable precipitation in the absence of NaCl. The added electrolyte also enhances the potential for partial adsorption of free SDS by lowering its CMC. This greatly increases the partial surface pressure of SDS which may also lower the surface tension peak. We examine the region of free SDS behavior in more detail below.

The surface tension peak may also occur in weak PE–S systems. PVPm is known to be about 50 and 40% ionized with and without 100 mM NaCl, respectively.^66^ In the absence of NaCl, SDS–PVPm exhibits a sharp increase in surface tension shown in Figure 7b, similar to that for SDS–PDDA. The peak occurs at a higher polymer concentration relative to SDS–PDDA, mainly because the polymer concentration is 7× higher than used for the SDS–PVPm. NR shows that the stoichiometry of the SC complex in NaCl is approximately *SP*. If the stoichiometry at the surface is also *SP* in the absence of NaCl, the similarity of the peak to that of SDS–PDDA, combined with the earlier arguments concerning the cause of the peak, suggest that the surfactant stoichiometry in the NE complex is higher than *SP*. This implies that the surfactant assists ionization and complexation in both bulk and surface complexes. However, the position of the peak with respect to *s*_*N*_ is different in the two systems, occurring well above *s*_*N*_ for PDDA but close to the sharp rise in surface tension for PVPm, i.e., a higher *relative* surfactant concentration is required to form the NE complex in SDS–PDDA than in SDS–PVPm in the absence of added NaCl. A further difference between the two systems is the weakness of the surface tension peak for SDS–PVPm in NaCl. This may result from a small change in tension if the stoichiometries of NE and SC complexes are similar and/or because the higher concentration used for SDS–PVPm shifts the peak to a concentration much closer to the onset of the CMC of the surfactant. The free surfactant may then be sufficiently surface active to cause a further lowering of the surface tension, which we discuss below.

### Surface Composition and the Butler Equation

**Figure 8:**
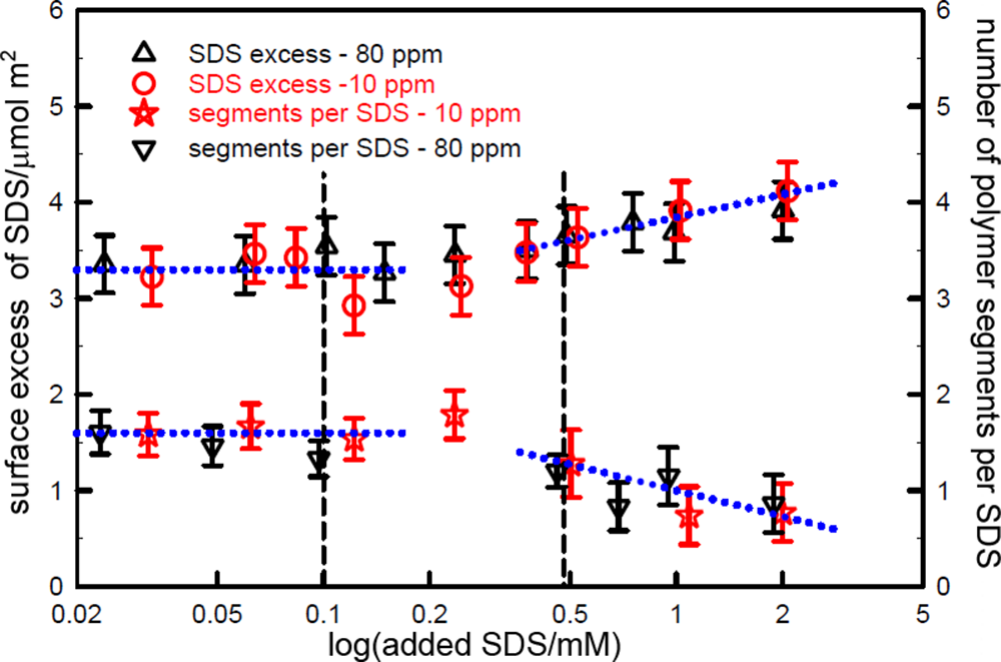
Variation of surface coverage of SDS and the number of polymer segments per adsorbed SDS with added surfactant for PDDA concentrations of 10 and 80 ppm in 100 mM NaCl. The fixed PDDA concentrations are marked by dashed lines. The data are redrawn from Staples et al.^6^ The dotted lines are guides to the eye.

The PE–S system has so far been treated as having a single SC surface active species, i.e., the Gibbs equation can be used to describe the surface behavior. However, *s*_free_ increases as more surfactant is added and its partial surface pressure, σ_free_, will eventually become comparable with that of the SC complex and hence either or both may contribute to the surface pressure and the system is then better described using the Butler equation.^52,53,70^ The onset of an increase in the surface tension signals a decrease the partial tension of the complex, σ_*C*_ (complex), while the steady increase in *s*_free_ increases the partial tension of the free surfactant, σ_free_. Free surfactant then starts to displace the complex. Surface composition data in Figure 8 show this change clearly for two compositions of the SDS–PDDA system in electrolyte. Below the onset of the surface tension peak only the *S*_0.5_*P* complex is adsorbed, as expected, but free SDS starts to adsorb above the surface tension peak as an increasing σ_free_ causes the complex to be gradually displaced from the surface, although some adsorbed complex still remains at compositions above the peak. Addition of electrolyte also lowers the CMC of the surfactant and hence also increases σ_free_ for the free surfactant.

**Figure 9:**
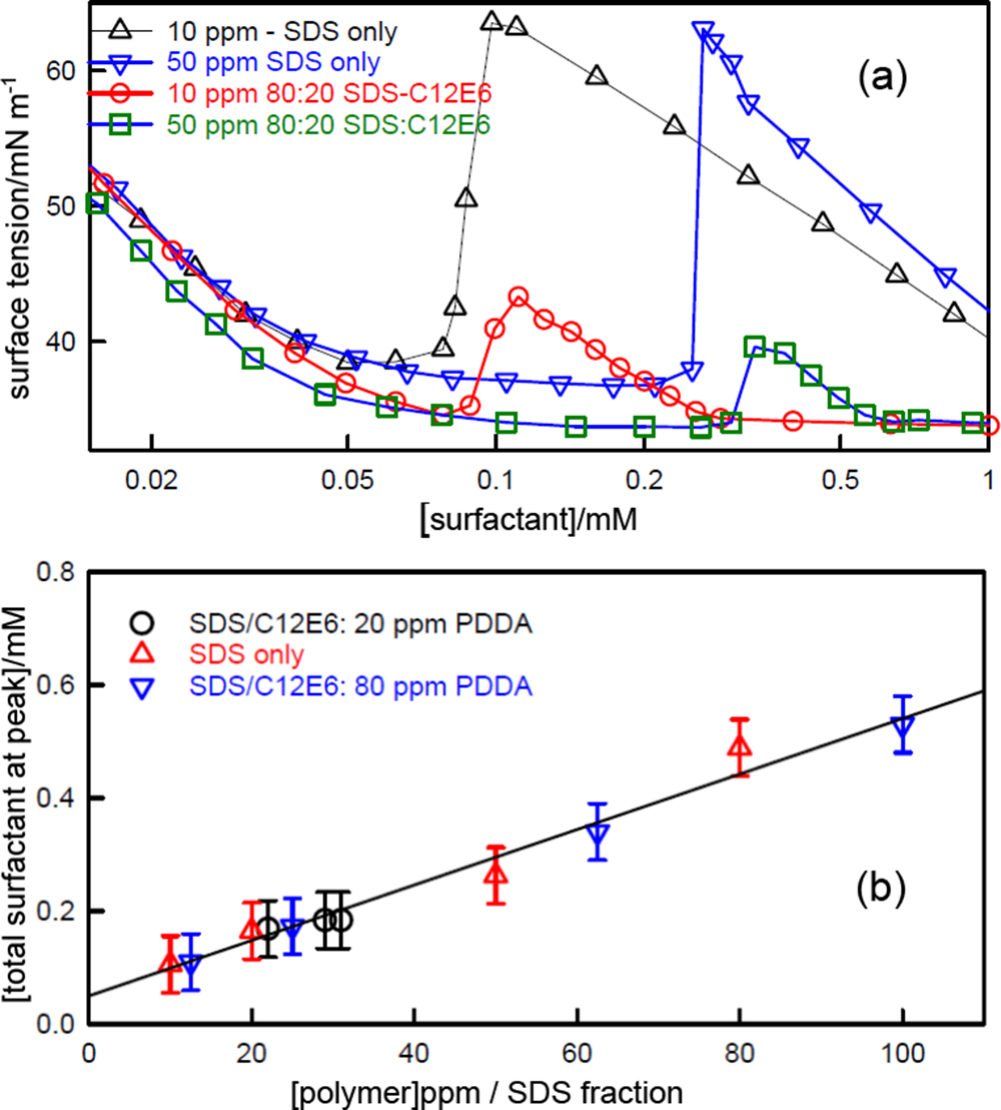
(a) Surface tension of 10 and 50 ppm PDDA in 100 mM NaCl as a function of added SDS only and of SDS/C_12_E_6_ mixtures, and (b) the variation of the total surfactant concentration at the surface tension peak as a function of SDS concentration in both the absence of C_12_E_6_ and in mixtures with C_12_E_6_. The data are from Staples et al.^7^

Further understanding of the role of the Butler equation in the SDS–PDDA system comes from experiments in which the nonionic surfactant hexa–ethylene glycol monododecylether (C_12_E_6_) is added to the SDS–PDDA–NaCl system. Addition of nonionic surfactant has a large effect on the surface tension behavior, as shown in Figure 9a, where the surface tension peaks change position and lose intensity as the fraction of added C_12_E_6_ increases.^7,71^ A plot of the positions of the peaks against segment:SDS ratio in Figure 9b shows that the peak positions depend only on the average charge in that all the points lie on the same straight line, with or without C_12_E_6_. This is consistent with the polymer binding to clusters of surfactants whose compositions do not affect the dominant charge interaction between surfactant and polymer, i.e., C_12_E_6_ does not interact with PDDA. The surface tension of C_12_E_6_–SDS mixtures is however very different from that of SDS alone.^31,72^ Thus, an SDS–C_12_E_6_ mixture maintains a low tension down to much lower concentrations than SDS. Independently of whether or not the C_12_E_6_ participates in the formation of the PE–S complex, the Butler equation requires that the higher surface pressure of the SDS–C_12_E_6_ combination causes it to displace the complex from the surface more completely than SDS on its own. This is clearly seen at and above the onset of the surface tension peak, where surfactant starts to compete strongly with PE–S complex for the surface, and the mixture therefore acts to suppress the surface tension peaks, as shown by the large drops in tension in Figure 9a.^7,71^ However, the effect is more clear in the behavior of the surface composition.

**Figure 10:**
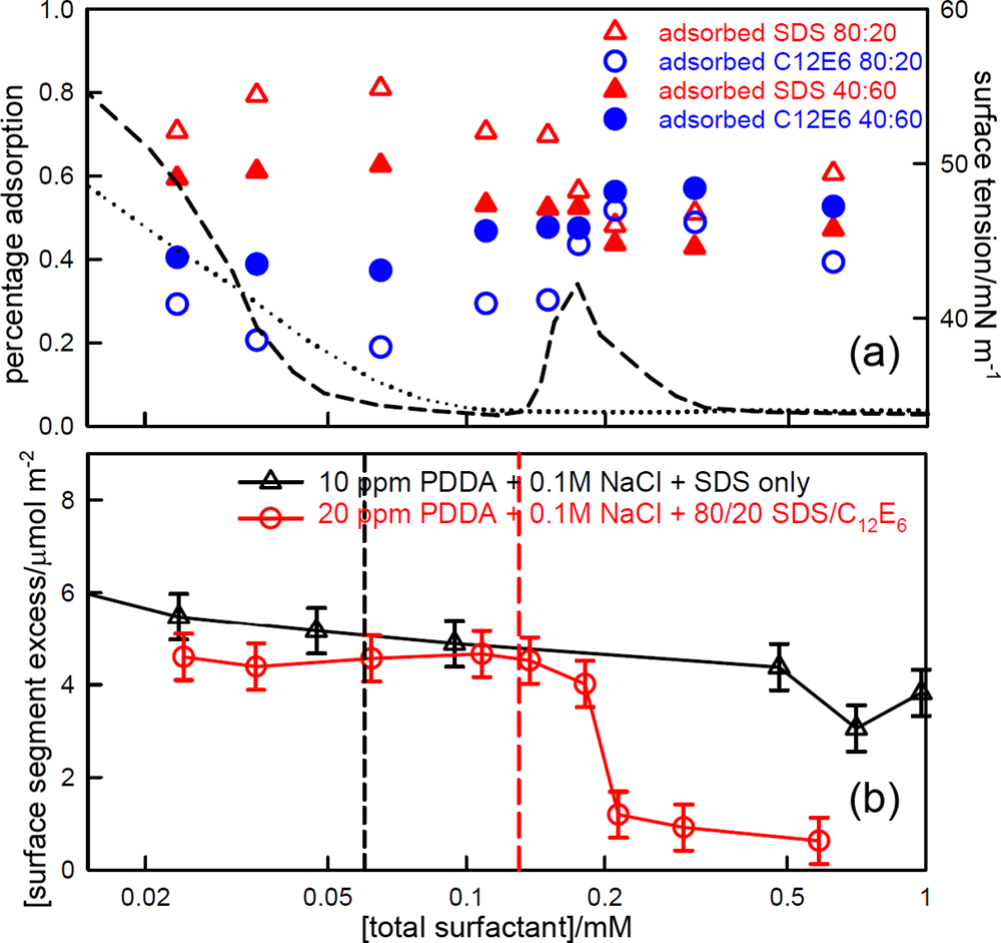
(a) Surface excesses of SDS and C_12_E_6_ in mixtures of 20 ppm PDDA with SDS:C_12_E_6_ ratios of 80:20 and 40:60 with 100 mM NaCl. Dashed and dotted lines are respectively the surface tensions of the 80:20 and 60:40 mixtures. (b) Comparison of the variation of the surface excesses of adsorbed PDDA segments in SDS and in SDS:C_12_E_6_ mixtures as a function of surfactant concentration. The charge equivalence points are marked by a dotted line for SDS alone and by a dashed line for the SDS:C_12_E_6_ mixtures. All the data are from Staples et al.^6,7^

SDS interacts attractively with C_12_E_6_ both in micelles and at the A–W surface.^31,72^ The mixed CMC in 100 mM NaCl and for SDS fractions less than about 0.6, lies in the same low concentration range of 0.02–0.07 mM where PDDA and SDS interact strongly. Figure 10a shows that the surface excesses at low concentration contain significant levels of C_12_E_6_ in 40:60 SDS:C_12_E_6_ mixtures, and that these decrease when the C_12_E_6_ fraction is reduced to 80:20. However, in the presence of the polymer, increasing the total concentration of surfactant from 0.02 to about 0.06 mM leads to a *decrease* in the C_12_E_6_ adsorption and an increase in SDS adsorption. This is the reverse of what occurs in the *absence* of PDDA.^31^ The anomalous increase in SDS adsorption can only be the result of its strong and independent interaction with PDDA. In terms of the Butler equation the surface pressure of the strongly interacting PDDA–SDS complex causes it to displace the more weakly adsorbing SDS–C_12_E_6_ combination. The drop in C_12_E_6_ adsorption also confirms that there is little interaction between PDDA and C_12_E_6_. Below an *s*_total_ of about 0.1 mM the bulk concentration of surface complex (*S*_0.5_*P*) starts to become depleted. In the absence of C_12_E_6_ the surface tension would then normally start to increase. However, because C_12_E_6_ is being steadily added and is not consumed by complex formation, the surface tension is maintained at a low value by the surface composition becoming increasingly rich in SDS relative to C_12_E_6_. C_12_E_6_ then becomes the dominant adsorbed species up to about 0.4 mM, reaching its maximum at and above the position of the normal surface tension peak.

The free SDS concentration is also expected to increase strongly after the bulk SP complex is formed because of its significant attractive interaction with C_12_E_6_. The similar final compositions of the two surfactants in the layer at about 0.6 mM are approximately consistent with the known nonideality of SDS–C_12_E_6_ mixtures above the mixed CMC. Thus, the overall effect of the added C_12_E_6_ is *not* that it solubilizes the mixture (precipitation has been shown to be similar to and without added C_12_E_6_^6,71^), but that it interferes with the surface behavior of SDS. A final interesting comparison is of the polymer adsorption with and without C_12_E_6_, which is shown in Figure 10b. In the presence of C_12_E_6_, polymer is more or less completely displaced from the surface when C_12_E_6_ is present, whereas in the absence of C_12_E_6_, polymer remains at the surface up to a total surfactant concentration an order of magnitude greater than that at the peak in the surface tension. That this is brought about by a higher surface pressure in the layer is also consistent with the presence of the strongly surface active C_12_E_6_.

### Surfactant Excess Region

Below *s*_*N*_, surfactant and polymer combine to form a PE–S complex that is more surface active than either individual component. However, at the onset of bulk NE complex formation and as further surfactant is added, this SC complex may either be converted to the non-surface active NE complex with free surfactant then becoming the dominant surface species, or it may evolve to a larger surfactant rich complex that may or may not remain a strong or dominant surface active species. The value of *s*_free_ typically changes from a low value at charge equivalence (*s*_*N*_) to a much higher value (≈ CMC) at the onset of formation of the AG complex. The *s*_free_ increase in this range may be one or 2 orders of magnitude. There are then two simple alternatives in the surfactant excess region. In the first, the PE–S complex effectively loses its surface activity and is progressively pushed from the surface by the increasing σ_*s*_ of the free surfactant, as happens in SDS–PDDA. In the second, the PE–S complex incorporates extra free surfactant to form a surfactant rich complex with a high enough partial surface pressure to remain the preferred adsorbate. In *strong* PE systems a simple indicator of whether such a surfactant–rich species might form is the relative stoichiometry of the SC and NE complexes. If an SC complex is deficient in surfactant relative to the NE complex it suggests that additional complexation of surfactant into the surface layer is unfavorable and this is what occurs in the SDS–PDDA system, where no surface active PE–S complexation is observed at higher surfactant concentrations. The situation in *weak* PE systems is, however, different because of the presence of uncharged and surface active polymer segments.

A surface structure that incorporates extra surfactant is the trilayer, which is formed by the attachment of a bilayer of surfactant to the aqueous side of the original PE–S layer.^8^ This is a commonly observed structure at the surface of mixtures of surfactant with multivalent ions, including polyelectrolytes.^9^ It also has a large effect on the NR signal and is therefore easy to detect. The formation of a bilayer is favorable if there is (i) a strong tendency for the surfactant to aggregate, i.e., *s*_free_ has reached a concentration at or above the usual CMC, and (ii) when the packing parameter for the surfactant favors a planar lamellar rather than a micellar structure. Leaving aside these two factors, the initial monolayer must obviously also be stable with respect to the addition of more surfactant. The first two factors can be judged to some extent by examining the effect of the surfactant chain length in trilayer formation.

**Figure 11:**
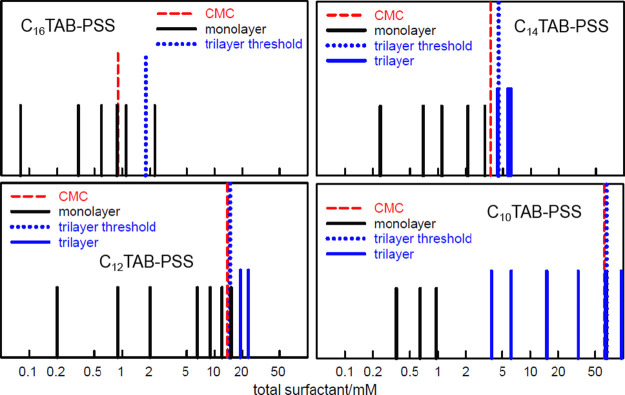
Patterns of trilayer formation at the A–W surface of mixtures of C_*n*_TABs with 20 ppm of NaPSS (0.1 mM segments). The shorter black vertical lines represent the mixed PE–S monolayer and the taller blue lines indicate trilayer adsorption. The nominal CMC is marked with a red dashed line and the concentration at which the *free* surfactant reaches the CMC is marked with a blue dotted line. The data are from Taylor et al.^68^

Taylor et al. have explored the formation of trilayers in the C_*n*_TAB–PSS system as a function of *n* and the results are shown in Figure 11.^68^ In this Figure, a vertical red dashed line marks the normal CMC and the blue dotted line marks the slightly higher concentration at which the *free* concentration of surfactant reaches its CMC. The black lines mark concentrations at which only monolayer adsorption is observed and the taller and thicker blue lines mark concentrations where a trilayer forms. The figure shows that trilayers form in C_12_TAB and C_14_TAB when *s*_free_ is above the CMC but do not form at all for C_16_TAB.^68^ However, trilayers start to form below the CMC for C_10_TAB. This pattern of behavior is then consistent with the trilayer stability being partly a result of more favorable aggregation above the CMC and partly a result of the increase in the packing parameter, which favors lamellar structures as the alkyl chain shortens. However, in a separate experiment on C_12_TAB–PSS with a PSS sample of *lower* MW (18k rather than 48k) the trilayer was also found to form at concentrations *below* the CMC, similarly to C_10_TAB–PSS with the higher MW PSS, but with distinct time fluctuations.^8^ The combination of these results suggests that both the onset of the CMC and a higher packing parameter assist trilayer formation. However, they also indicate that the MW of the polymer may have a significant effect, with trilayer formation being favored by smaller MW species. At the fringes of stability of the trilayer structure, the system is evidently sensitive to polydispersity of the MW. Hence the time–dependence in the lower MW sample.

The surface tension of C_12_TAB–PSS forms a low plateau following its initial drop and this plateau is maintained up to another small decline as *s*_free_ approaches the CMC (Figure 2b). However, in the corresponding concentration range, C_14_TAB–PSS and C_16_TAB–PSS have surface tension peaks, less sharp but similar to those seen for SDS–PDDA except that the C_*n*_TAB peaks occur at higher concentration and are clearly caused by the onset of AG complex formation, which now depletes the surface active species in the same way that the NE complex depletes the SC complex in SDS–PDDA. Taylor et al. did not observe such a peak for C_12_TAB–PSS in either of their PSS samples of different MW. However, Varga and Campbell (VC) did obtain a peak for their C_12_TAB–PSS and have attributed the discrepancy to a failure by Taylor et al. to reach equilibrium.^43^ However, Taylor et al. used research grade PSS that was close to completely sulfonated (100% for MW 18k and 97% for 48k), whereas VC used a commercial grade PSS, with the standard 80% sulfonation. The incomplete charge in the commercial grade is a consequence of the post–polymerization sulfonation used to make commercial grade PSS (free radical polymerization of the styrenesulfonate is required to achieve the near complete sulfonation of the samples used by Taylor et al.).^45^ Apart from a significantly incomplete sulfonation, post polymerization of PS leads to the formation of hydrophobic loops in the structure, which will cause the commercial polymer to be significantly more, or differently, surface active than the polymer prepared by presulfonation.^45,46,73^

### Equilibration and Polydispersity

Although much attention has been given to the potential for mixing procedures to lead to non-equilibrium structures in PE–S systems, little attention has been given to how either MW or composition polydispersity might couple to the patterns of surface and/or bulk behavior in PE–S systems. The present analysis shows that it is possible to account at least semiquantitatively for the surface behavior of dilute PE–S systems within the framework of equilibrium thermodynamics with the implied assumption that the polymer is monodisperse. However, polymers are polydisperse and, as is clear from the discussion in the previous section, it is likely that this will affect the surface behavior, especially if its charge is polydisperse. Because the Gibbs equation depends on molar quantities, the analysis of any effects of polydispersity in polymer systems is particularly intractable^19^ and there have been few attempts to address the issue.^20,21^ The simplest empirical approach is to measure the surface tension curves of different MW polymers and then to examine the consistency of each with the Gibbs equation. This has essentially been done several times on the nonionic poly(ethylene glycols), most recently by Gilanyi et al.,^74^ who found that the optimum measurement time was about 1 h, i.e., neither too fast nor too slow. An et al.^21^ came to a similar conclusion using NR to analyze the apparently anomalous surface tension behavior of a relatively monodisperse and low MW poly(vinyl methyl ether). The range of systematic time measurements that have been made on PE–S systems is extensive and offers the possibility of using these to obtain a better understanding of some of the equilibration processes.

The initial MW composition of any polymer aggregate formed in solution or at the A–W surface is expected to be the sample average because there is not time for spatial rearrangement across the solution. Any optimization of MW or charge composition of aggregates can then only be reached by subsequent slow exchange of species of different MW or charge with the bulk solution. The low *molar* concentration of individual species will make this process very slow. Thus, any preferential segregation in an aggregate in either the surface layer or the bulk complex will cause the surface tension to change on a time scale characteristic for that particular equilibration. Two situations where this possibility can be expected are the low concentration plateau associated with counterion condensation and the surface tension peak associated with depletion by NE complex formation in the SDS–PDDA and SDS–PVPm systems.

In the absence of added electrolyte, counterion condensation causes the bulk activity of the PE to be constant over a range of low surfactant concentration. The Gibbs equation then requires the surface tension to remain at its value for pure water even if a layer of polymer is present at the surface, as is observed for all three systems in Figure 2. However, Bohme and Scheler^75^ have shown that counterion condensation progressively disappears as the MW of the polyion falls to that of the monomer, i.e., the solution activity of small polyions increases with molar concentration rather than remaining approximately constant. Small ions (oligo- rather than poly ions) therefore have a higher surface activity. At low concentrations where counterion condensation occurs, the initially adsorbed polymer layer at the A–W interface will tend to have an average MW distribution, i.e., the surface tension remains high because counterion condensation causes the average MW species to have an constant low activity. However, the small MW species have a higher activity because they do not undergo counterion condensation. They are therefore more surface active and will gradually displace the large MW species and cause the surface tension to decrease. At low concentrations, all three systems in Figure 2 have the same tension as pure water over the usual time scale of such measurements, even though there is substantial adsorption, as shown for SDS–PDDA by both NR^63^ and sum frequency generation.^65^ However, for all three systems in Figure 2, the initial surface tension plateau is replaced by a steady drop over about 4–5 h.^10,69,76^ An earlier explanation that attributed the slow equilibration to a charge barrier^77^ is not consistent with the coexistence of a complete adsorbed layer and a high surface tension.

In the vicinity of the surface tension peak of SDS–PDDA, precipitation of the NE complex occurs and can again be expected to be relatively non-selective in the initial precipitation step. However, smaller MW species would be expected to be more soluble than large MW species, partly because of their greater solution entropy. They will therefore tend to leach out from the precipitate, and then effectively act as a reservoir of surface active species that can restore the low surface tension and hence suppress the peak. This dissolution process would be expected to be significantly slower than the counterion condensation exchange described above because the surface active species are trapped in a more dense aggregate. Consistent with such a mechanism, Noskov et al. have found that the surface tension peak from precipitation of SDS–PDDA remains for 11 h before disappearing,^10^ compared with only 4 h for the counterion condensation plateau to disappear. Similarly, for SDS–PVPm without electrolyte the initial surface tension plateau had disappeared at 5 h but the peak was still present after this time.^78^ In the presence of electrolyte, Figure 5a shows that SDS–PDDA also has a significant peak associated with precipitation at higher concentration. That this is fully reproducible over the typical 1 h time scale is demonstrated by the close agreement of the surface tension peak observed by Varga and Campbell^43^ with the earlier results of Staples et al.^6^ However, measurements by Lyadinskaya et al.^79^ show that this peak disappears over about 5 h. This must be caused by the same mechanism as in the absence of electrolyte, but it is significantly faster, probably because the presence of electrolyte generally enhances rates of solution of electrolytes.

### Weak Polyelectrolytes

The complication in the surface tension behavior of weak PE–S systems is that the surface behavior may be determined by the binding of either surfactant–ionized segment pairs (≈eq 5), and/or an uncharged segment (≈eq 2). Ionization of segments may also be enhanced by interaction with a more polar headgroup in the surfactant, such as -SO_4_^–^ or -SO_3_^–^, or suppressed by a less polar headgroup, such as R(CH_3_)_3_^+^. Such effects are expected to be different for bulk and surface complexes. However, the main difference expected in weak PE–S systems is that the initial high surface tension plateau caused by counterion condensation will often disappear, SDS–PVPm being an exception. For the SDS–PVPm measurements in Figure 2c, the PVPm was fully quaternized and any hydrolysis to give free electrolyte was evidently low enough not to interfere with counterion condensation. The remaining surface tension behavior of SDS–PVPm can also be described in terms of the strong PE–S model used for the two strong PE–S systems considered above. In the following, we consider three systems where weak electrolyte behavior is more evident.

If there is no counterion condensation, the surface tension should drop steadily to an approximate plateau, as originally demonstrated for the weak PE–S system SDS–poly(L–lysine)(PLL) by Buckingham et al.^14^ The surfactant concentrations at the start and finish of this plateau can be defined in terms of the binding curve. In principle, this can also be done for a weak PE–S system but the binding curve itself varies with pH. Binding curves at different pH have been determined for C_14_TAB–PAA in 10 mM NaBr by Kiefer et al.,^61^ while surface tension and NR measurements on the closely related C_12_TAB–PAA without added electrolyte have been made by Zhang et al.^80^ Hayakawa et al.^81^ have further shown that for this particular case and these conditions, the CACs of the two surfactants are approximately the same because the increase in the CAC of the C_14_TAB system caused by added electrolyte approximately cancels the decrease from the difference in chain length. This allows a useful comparison of the binding and surface properties of C_14_/C_12_ to PAA.

**Figure 12:**
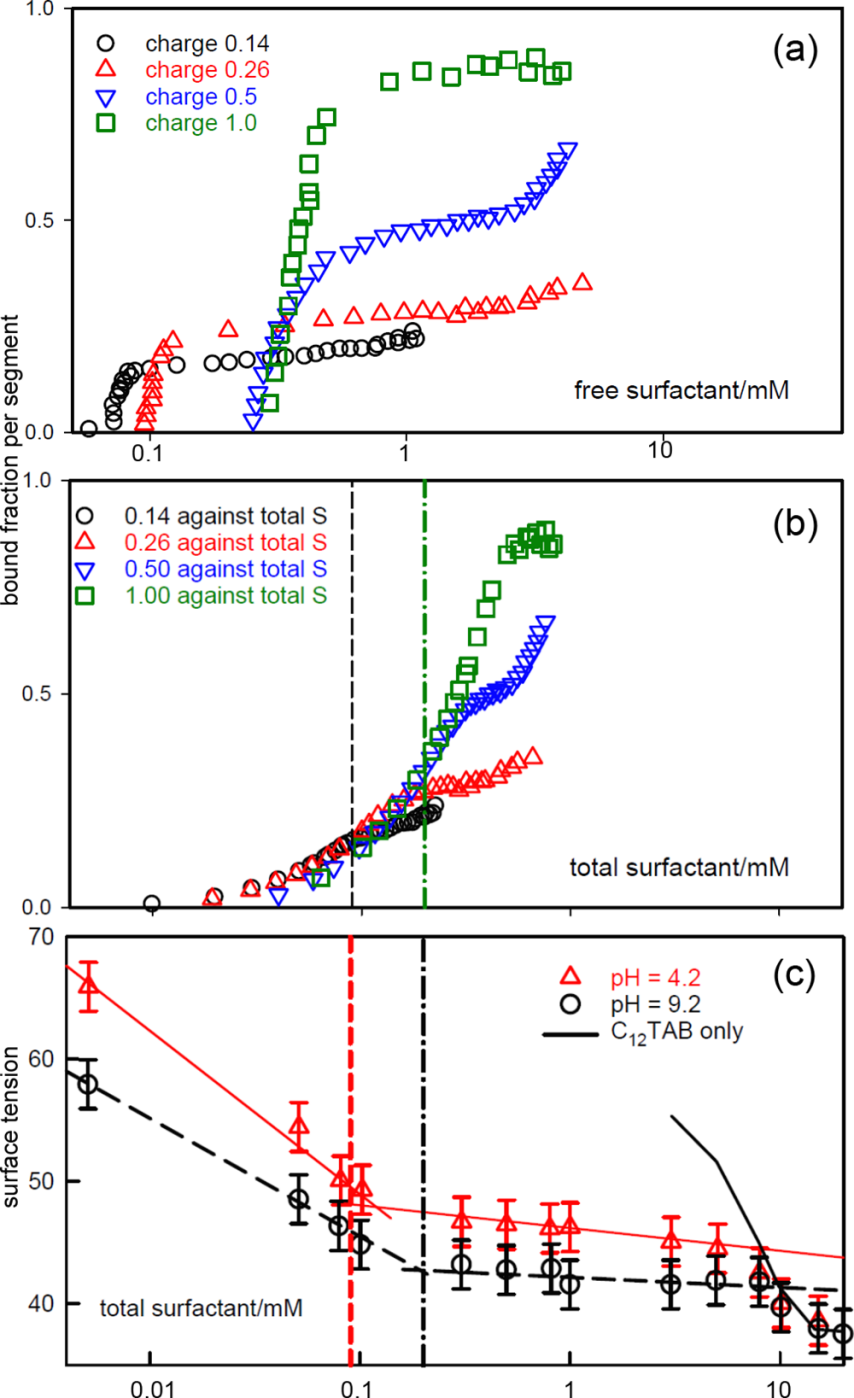
Surfactant electrode data for C_14_TAB/PAA and surface tension data for C_12_TAB/PAA. (a) Bound fractions of C_14_TAB per PAA segment as a function of *free* C_14_TAB, (b) the same bound fraction of C_14_TAB per PAA segment as a function of *total* C_14_TAB, and (c) the surface tension of C_12_TAB at two pH (4.2 and 9.2) corresponding to ionized fractions of PAA of approximately 0.1 and 0.9. The data in (a,b) are redrawn from Kiefer et al.,^61^ and those in (c) are from Zhang et al.^80^

Figure 12a plots the fractional binding per charged segment against *s*_free_ for C_14_TAB–PAA as obtained by Kiefer et al.^61^ For a comparison with the surface tension and NR experiments, these results are replotted as fractional binding per segment against *s*_total_ in (b). The vertical lines in (b) mark points at about 25% cooperative binding for the lowest (0.14) and highest (1.0) fractional charged states of PAA. Figure 12c plots the tension of C_12_TAB–PAA at pH values corresponding to charged fractions of 0.1 (pH = 4.2) and 0.9 (pH = 9.2) against *s*_total_,^80^ which are the closest to those of 0.14 and 1.0 in the electrode measurements of (a) and (b). The surface tension shows the initial decrease expected from the lack of counterion condensation, up to an onset of the central near–plateau, taken to correspond to a bound fraction of about 25%. This is followed by the more gradual change in tension expected in the cooperative range of binding. NR shows directly that binding is already significant at 0.001 mM with surface excesses of surfactant of 2.1 and 1.9 ± 0.2 μmol m^–2^ at pH = 4.2 and 9.2 respectively, which increase to 3.6 ± 0.2 μmol m^–2^ for both pH at 1 mM, close to that for the normal monolayer at the CMC of 14 mM.^82^ NR measurements also show that, at an *s*_total_ of 0.1 mM, the surface layer consists of approximately 3 PAA segments to one surfactant *for both* pH. This suggests that the surface contains an (*SP*) ion pair at both pH but the remaining two PAA segments are mainly in their nonionized acid form at pH 4.2 but are partially ionized at pH 9.2.

In strong PE–S systems a low relative stoichiometry of surfactant to segment was taken to be an indication that the surface is susceptible to depletion in the vicinity of NE complex formation. In SDS–PDDA this leads to an surface tension peak because the (*SP*_2_) surface complex effectively becomes unstable with respect to an increase in *s*_total_. The reason is not known but it probably results from a conformation of the *S*_0.5_*P* unfavorable to the addition of an extra surfactant molecule either in the upper (air) layer or as a start of an underlying bilayer. In the weak C_12_TAB–PAA system with *S*_0.3_*P* stoichiometry, there is significant extra space to adsorb at least one extra surfactant and, at a pH of 9.2, the negatively charged layer strongly favors further adsorption. It is then not surprising that the extra adsorption is in the form of a bilayer attached to the original monolayer, i.e., a trilayer, but only at the high pH. Following the discussion of the conditions that favor trilayer formation above, this occurs only at the higher concentrations of 3, 10, and 20 mM, where surfactant aggregation is generally more favored.

**Figure 13:**
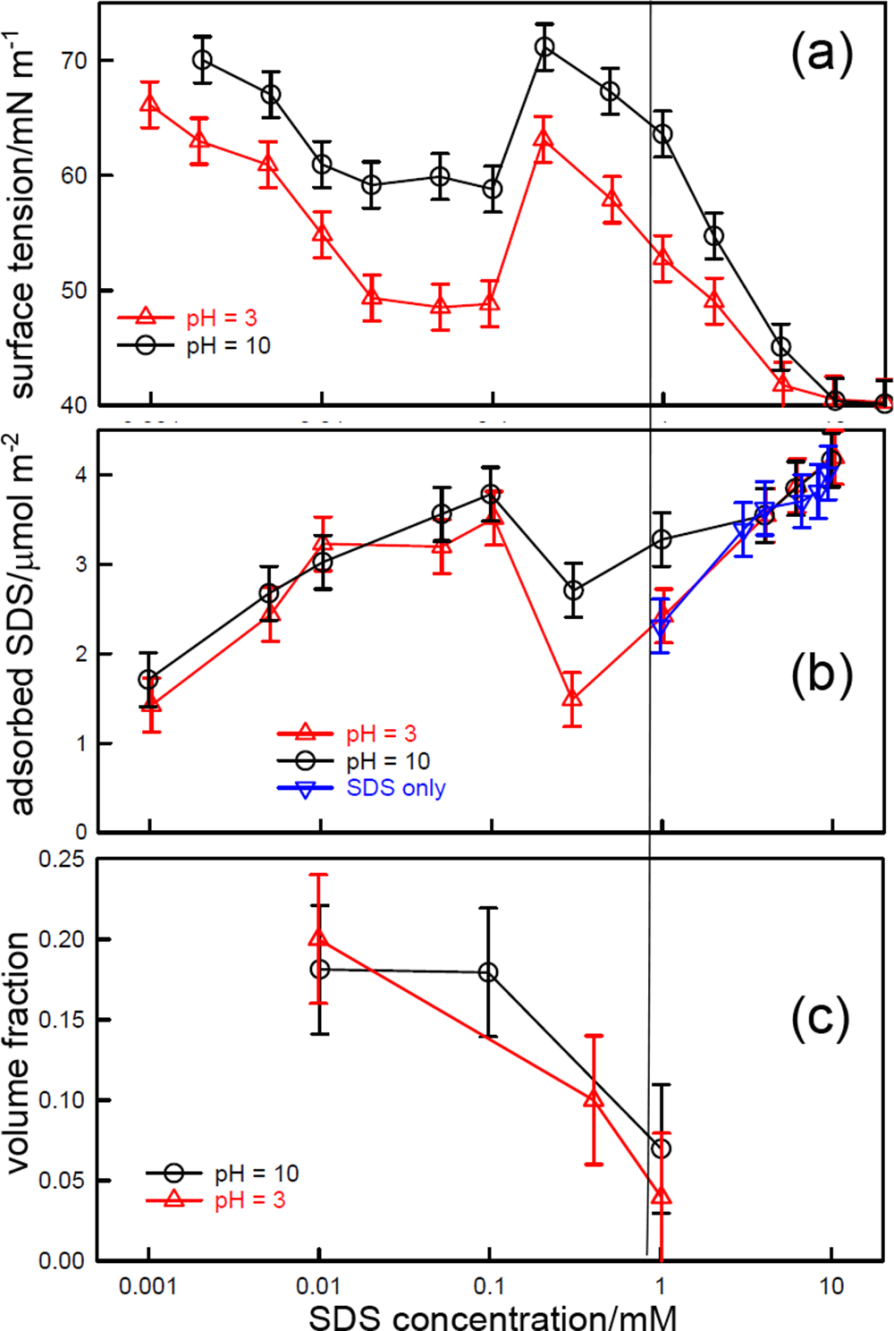
Surface tension and adsorption data for SDS–PLL at pH values of 3 and 10, (a) surface tension of 20 ppm PLL (0.9 mM) as a function of added SDS, (b) surface excess of SDS at the A–W surface, and (c) the volume fraction of PLL in the surface layer. The data are from Zhang and co-workers.^83^ The thin vertical line marks surfactant–segment equivalence.

Whereas the surface is stabilized by the addition of more surfactant to the layer in C_12_TAB–PAA, the opposite occurs in the two other weak linear PE–S that have been investigated by NR, SDS–PLL, and SDS–PEI. In these systems an increase in surface tension occurs as the mean activity of the surface complex is depressed by the formation of the bulk NE complex. Figure 13a shows the surface tension behavior of SDS–PLL at pH values of 3 and 10 in the absence of electrolyte.^83^ There is the expected initial gradual drop and then a sharp increase in tension at about 0.2 mM below *s*_*N*_. Figure 13b shows that the SDS surface excess initially steadily increases before there is a sharp dip in the surface excess, shortly after which the SDS surface excess increases further as polymer is pushed off the surface (Figure 13c). The pattern is qualitatively similar to that of SDS–PDDA. The composition of the surface complex is approximately (*SP*) with a slight excess of surfactant at the low pH and a slight depletion at pH 10. The conformation of the protein is different at the two pH with a higher solubility at the low pH. The depletion at pH 10 is therefore also very similar to that in SDS–PDDA. The data shown in Figure 13 is for a 58k MW sample. The upturn in the surface tension was also observed by Buckingham et al. but only for high MW material (68.5k) not for a low MW (4800). Similar patterns of a surface tension peak, have been obtained for SDS with linear poly(ethylene imine), but only when the polymer is linear, and the NR measurements indicate a less marked variation in the adsorption than in the SDS–PLL system(28k).^84^

## Conclusions

In a previous paper we showed that data from binding isotherms with surface tension and independent measurements of surface excess at the A–W interface are broadly consistent with the Gibbs equation for two particular *strong* linear PE–S systems.^30^ Thermodynamic consistency, although an approximate average and limited to two systems, is a more definitive test of equilibration than empirical observations of the time behavior. In the present paper we extend the thermodynamic analysis by including (i) *weak* PE–S systems, (ii) the requirements of the Butler equation for mixed surface active species, (iii) an extension of the upper concentration range up to the onset of multilayer adsorption, and (iv) some kinetic consequences of the polydispersity of the polymer.

At the lowest accessible surfactant concentrations the surface tension behavior appears to be dominated by counterion condensation, which causes the tension to be little different from the high value of water because the mean activity of the PE–S complex is low. Thus, at the onset of the addition of surfactant there is a range of concentration where surfactant ion may become part of the condensate. If this extends to where surfactant ion starts to bind cooperatively (the CAC) the surface tension remains high until cooperative binding is complete. For this to occur, the separation of charge in the PE must be less than the Bjerrum length, which occurs for the strong PE–S and the weak SDS–PVPm, but not for the other weak PE–S systems. This surface tension plateau is also noticeably weakened if the surfactant is not strongly hydrophobic, e.g., SC_10_S-PDDA. Strong PE of *low* MW are not expected to exhibit counterion condensation and are therefore more surface active than large MW species. Polydispersity may therefore be an explanation of the known very gradual drop of the surface tension with time for dilute strong PE–S systems.

The surface tension reaches a low approximate plateau in the range between completion of *cooperative* binding and the formation of a near equivalent (NE) complex, which may be either an insoluble precipitate or a soluble phase/pseudophase. The main complication in understanding the surface behavior in this concentration range is that the adsorbed layer, i.e., the surface complex (SC), often has a different stoichiometry from the bulk NE complex. If the SC complex contains a lower fraction of surfactant than the NE complex, the concentration of the SC complex may decrease as NE complex is formed. If there is a gap between the loss of the SC complex and a buildup of sufficient free surfactant to maintain a low surface tension, there will be a peak in the tension. A higher fractional content of surfactant in the SC complex indicates that it is more surface active than the NE bulk complex and it will therefore continue to occupy the surface even if the NE complex is precipitated. Further addition of surfactant may lead to the formation of either a surfactant rich surface active complex, such as a trilayer, or to a greater contribution of free surfactant to the surface tension of a mixed surface layer. In both cases the surface tension will remain low, although the composition of the layer will not be the same. The factors that favor formation of a trilayer are those that favor aggregation, i.e., an *s*_free_ above the usual CMC and a packing fraction that favors lamellar structures. The occurrence of a trilayer is also sensitive to MW and fractional charge on the polymer.

The competition of the two surface active species (free surfactant and SC complex) leads to two interesting phenomena. First, the effects of the addition of a *non-ionic* surfactant, which interacts strongly and in a known way with just the charged surfactant, are consistent with the requirements of the Gibbs and Butler equations. Second, polydispersity may become important when precipitation of the NE complex occurs. Thus, initial precipitation is expected to be non-selective with respect to MW because smaller species become trapped. However, smaller species of the NE complex are expected to be more soluble than larger species and will tend to leach out over long times. Their intrinsically higher solubility makes them more surface active than the larger species in the precipitate and they therefore act to maintain a low surface tension in the range where there is a high tension peak. The peak will then disappear over a long time period, as observed.

The complication of *weak* PE–S systems is that undissociated polymer segments have a tendency to be surface active. Adsorption of a PE–S complex may then be driven not just by surfactant ion–dissociated segment interactions but by uncharged segments and/or by surfactant ion induced ionization of segments at the surface. The extent to which either effect occurs is expected to be different between bulk solution and surface. The approximate quantitative treatment of the surface tension used for strong PE–S systems is then only applicable if the surfactant induces ionization of most of the segments. This seems to be the situation for the SDS–PVPm system, whose surface tension behavior is similar to those of the two strong PE–S systems studied here.
